# Counting growth factors in single cells with infrared quantum dots to measure discrete stimulation distributions

**DOI:** 10.1038/s41467-019-08754-5

**Published:** 2019-02-22

**Authors:** Phuong Le, Sung Jun Lim, Brian C. Baculis, Hee Jung Chung, Kristopher A. Kilian, Andrew M. Smith

**Affiliations:** 1 Department of Bioengineering, University of Illinois at Urbana-Champaign, Urbana, IL 61801 USA; 2 Micro and Nanotechnology Laboratory, University of Illinois at Urbana-Champaign, Urbana, IL 61801 USA; 3 Intelligent Devices and Systems Research Group, DGIST, Hyeonpung, Daegu, 42988 Republic of Korea; 4 Department of Molecular and Integrative Physiology, University of Illinois at Urbana-Champaign, Urbana, IL 61801 USA; 5 Neuroscience Program, University of Illinois at Urbana-Champaign, Urbana, IL 61801 USA; 6 School of Materials Science and Engineering, University of New South Wales, Sydney, NSW 2052 Australia; 7 School of Chemistry, University of New South Wales, Sydney, NSW 2052 Australia; 8 Australian Centre for NanoMedicine, University of New South Wales, Sydney, NSW 2052 Australia; 9 Department of Materials Science and Engineering, University of Illinois at Urbana-Champaign, Urbana, IL 61801 USA; 10 Carle Illinois College of Medicine, Urbana, IL 61801 USA

## Abstract

The distribution of single-cell properties across a population of cells can be measured using diverse tools, but no technology directly quantifies the biochemical stimulation events regulating these properties. Here we report digital counting of growth factors in single cells using fluorescent quantum dots and calibrated three-dimensional deconvolution microscopy (QDC-3DM) to reveal physiologically relevant cell stimulation distributions. We calibrate the fluorescence intensities of individual compact quantum dots labeled with epidermal growth factor (EGF) and demonstrate the necessity of near-infrared emission to overcome intrinsic cellular autofluoresence at the single-molecule level. When applied to human triple-negative breast cancer cells, we observe proportionality between stimulation and both receptor internalization and inhibitor response, reflecting stimulation heterogeneity contributions to intrinsic variability. We anticipate that QDC-3DM can be applied to analyze any peptidic ligand to reveal single-cell correlations between external stimulation and phenotypic variability, cell fate, and drug response.

## Abstract (web summary)

Measuring growth factors in single cells at physiologically relevant stimulation doses is challenging. Here the authors use fluorescent quantum dots and calibrated three-dimensional deconvolution microscopy to digitally count growth factors in single cells and reveal stimulation distributions in cancer cells.

## Introduction

Single-cell analytical techniques are reshaping our understanding of biology by revealing the distribution of gene expression and phenotype across a population of cells^1,2^. Applied together with systems biology models and information theory, it is now becoming clear that any population of genetically identical cells naturally exhibits substantial cell-to-cell variability that is integral to the emergence of ensemble biological functions^3^. This heterogeneity has important consequences, as rare cells, rather than cells near the ensemble mean, often dominate clinically meaningful pathogenic processes and drug resistance^4–6^. However, a void exists in experimental techniques to measure how cellular decision-making processes underlying population variability derive from extracellular biochemical signals, such as peptide growth factors and cytokines^7,8^, which cannot be easily measured at the single-cell level. Biochemical stimulation, the induction of an intracellular biochemical signal (e.g., receptor activation and translocation) by binding of an exogenous biochemical factor, is usually inferred indirectly from the resulting change in gene expression or cell phenotype^8^. Moreover, input factors are typically applied at stimulation extremes (zero and near saturation)^9^, whereas physiologically relevant tissue concentrations are in intermediate regimes (*c* ~ 1–100 pM)^10,11^ over which cells exhibit sensitive and heterogeneous dose–response relationships (EC_50_ ~ 1–100 pM)^12,13^. At these concentrations, relevant tissue microdomain volumes (~10 pL) contain just tens to hundreds of factors^14,15^, such that signal stimulation is temporally and spatially stochastic^16^. Accurate quantification of initiating signals is therefore very challenging^17^ and requires single-molecule sensitivity.

Here we describe a technology platform to digitally count growth factors in single cells using fluorescent quantum dots (QDs) and calibrated three-dimensional (3D) deconvolution microscopy (QDC-3DM). As a prototypical example, we focus on epidermal growth factor (EGF) and EGF receptor (EGFR)-positive cells. Fluorescent QDs are used as tags for EGF due to their extremely high fluorescence intensity that is homogeneous and stable at the single-QD level^18^. For maximum signal detection and comprehensive counting of EGF with rapid image acquisition, wide-field excitation is used to collect complete 3D images of cells, and deconvolution is used to reassign photons to their originating focal volumes. We observe that this methodology is only accurate when applying QDs with infrared emission due to interfering fluorescence from cellular components across the visible spectrum. We apply QDC-3DM to analyze EGF-induced cell signaling variability in triple-negative breast cancer cells (MDA-MB-231) grown on micropatterned islands to spatially register signaling events across separate cells. Our results show proportionality between stimulation and both receptor internalization and inhibitor response, reflecting stimulation heterogeneity contributions to intrinsic variability at the single-cell level.

## Results

### Imaging and image analysis

**Fig. 1:**
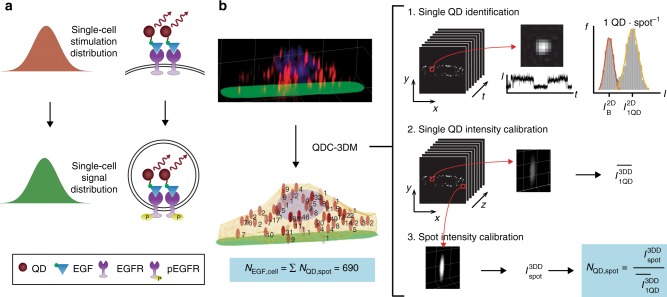
Quantum dot (QD) calibrated three-dimensional (3D) deconvolution microscopy (QDC-3DM). **a** Schematic representation of the contribution of single-cell stimulation distribution (growth factor binding) to signaling response distribution (measured by receptor internalization). **b** Depiction of the QDC-3DM image analysis methodology to count growth factors in single cells. The process begins with acquisition of 3D fluorescence images of single cells to localize single QDs and spatially register their locations. A representative 3D image shows a cell stimulated with QD-epidermal growth factor (QD-EGF) (red) on an Alexa Fluor 488-labeled fibronectin substrate (green) with nucleus labeled with Hoechst (blue). Each 3D image is deconvolved and spatially correlated to two-dimensional (2D) videos in the QD color channel. In the first step shown at right, time traces of spot intensities are used to identify single QDs by their distinctive two-component intensity distributions. In the second step, the average intensity of these single QDs from 3D deconvolved images, $\overline {I_{1{\mathrm{QD}}}^{3{\mathrm{DD}}}}$, is measured. In the third step, the 3D intensity of each spot, $I_{{\mathrm{spot}}}^{3{\mathrm{DD}}}$, is measured and registered to the average single-QD intensity, to calculate the number of QD-EGF per spot, *N*_QD,spot_. The number of EGF per cell, *N*_EGF,cell_, is then calculated as the sum of all *N*_QD,spot_

Figure 1a shows the overarching approach to measure the distribution of stimulation events of growth factors binding to cognate receptors, yielding a response distribution that plays an important role in the variability of signals and behavior between cells. Figure 1b summarizes the imaging and analysis methodology to measure absolute counts of growth factors, using two sequentially collected image stacks. A deconvolved high-resolution 3D epifluorescence image of cells is collected in three colors to distinguish QD-EGF conjugates (in red) spatially registered to the cell location by its fibronectin matrix (in green) and nucleus (in blue). The second image stack is a high temporal resolution video in the QD-EGF color channel. As described in detail in Methods, a three-step process is applied to count EGF molecules per cell: (1) Single QD-EGF spots are identified in videos by distinctive time-course intensity traces, *I*(*t*), for which two discrete intensities are present in two-dimensional (2D) images, $I_{1{\mathrm{QD}}}^{2{\mathrm{D}}}$ and $I_{\mathrm{B}}^{2{\mathrm{D}}}$, respectively, corresponding to the intrinsic QD intensity and its background due to on-and-off intermittency of emission (i.e., blinking)^19,20^. (2) Volumetric intensities of single QDs from deconvolved 3D images are averaged to yield $\overline {I_{1{\mathrm{QD}}}^{3{\mathrm{DD}}}}$, the average intensity of a single QD-EGF. (3) The number of contributing QDs to each spot in 3D images, *N*_QD,spot_, is calculated by dividing the volumetric spot intensity by the single-QD intensity. Finally, the total number of QDs is then calculated across each cell to determine the number of EGF per cell, *N*_EGF,cell_:

1 $$N_{{\mathrm{EGF}},{\mathrm{cell}}} = \mathop {\sum }\limits_{{\mathrm{spots}}} N_{{\mathrm{QD}},{\mathrm{spot}}} = \mathop {\sum }\limits_{{\mathrm{spots}}} I_{{\mathrm{spot}}}^{3{\mathrm{DD}}} \cdot \overline {I_{1{\mathrm{QD}}}^{3{\mathrm{DD}}}} ^{ - 1}.$$

### Quantum dot probe engineering

Accuracy of Eq. (1) requires that each QD bound per cell corresponds to a single EGF, thus requiring that each QD is bound to a single EGF. Monovalency between QDs and growth factors is further important to prevent artificial cross-linking between receptors that would not reflect the intrinsic monomeric nature of EGF. We optimized QD-EGF conjugates to ensure functional monovalency using an EGF engineered with a single N-terminal biotin, which self-assembles with covalent QD conjugates of streptavidin (SAv) with near covalent bond strength^21^.

**Fig. 2:**
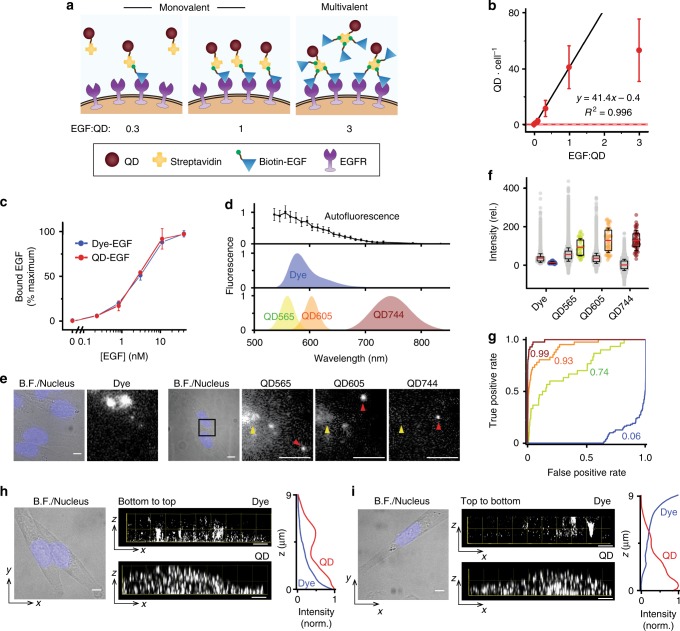
Functional and optical characterization of epidermal growth factor (EGF) conjugates of dyes and quantum dots (QDs). **a** Schematics show assembly of QD-EGF conjugates with controlled valency through biotin-streptavidin conjugation. **b** Experimental relationship between QDs bound per cell versus EGF:QD conjugation ratio. The red dashed line indicates the average number of QDs per cell for QDs alone, with red shade indicating the background standard deviation. MDA-MB-231 cells were treated with QD-EGF conjugates at 0.5 nM for 10 min on ice. *N* ≥ 10 cells. **c** Specific binding isotherm for dye-EGF and QD744-EGF to MDA-MB-231 cells measured by flow cytometry. Raw data are shown in Supplementary Figure 3. *N* = 2. **d** Fluorescence spectra of dye and QDs used in this work and mean autofluorescence, in arbitrary units. For autofluorescence, *N* ≥ 13 cells. **e** Representative images of autofluorescence, dye-EGF, QD565-EGF, QD605-EGF, and QD744-EGF bound to cell, measured in their respective spectral bands. All QD-EGF were bound to the same cell. The black square in each brightfield (BF)/nucleus micrograph indicates the zoom-in area shown in the fluorescence images. Yellow arrows indicate autofluorescence; red arrows indicate dyes or QDs. **f** Two-dimensional intensities of autofluorescence, single dye and single QDs. The box indicates 25/75th percentile; red lines are the mean value; whiskers are s.d.; *N* ≥ 30 for dye and QDs; *N* > 20,000 spots for autofluorescence at each wavelength. **g** Receiver operating characteristic (ROC) curve showing higher detection accuracy of single QD744 (dark red) compared with dye (blue), and QD565 (green) or QD605 (orange) in the presence of autofluorescence shown in **f**. Numbers indicate areas under the ROC curves (AUROC). **h** Representative BF/nucleus micrograph with orthogonal 3D fluorescence images of dye-EGF and QD744-EGF bound to the same cell, imaged from the bottom of the cell to the top. A z-projection of summed fluorescence intensity of dye-EGF and QD-EGF is shown at right. **i** Same as **h**, but fluorescence images were acquired from the top of the cell to the bottom. In **b**-**d**, points indicate mean ± s.d. In **e**, **h**, and **i**, scale bars, 5 μm

We adopted a previous strategy to generate monovalent conjugates by tuning the ratio between QDs and EGF (Fig. 2a)^22^ and used a functional assay to count the number of QD-EGF conjugates per cell. The discrete number of QDs bound to cells followed a linear trend with increasing EGF conjugation up to a 1:1 ratio, at which point multiple EGFs per QD no longer proportionally increased the number of QDs bound (Fig. 2b). We thus used an EGF:QD ratio of 0.3 to ensure that we were within the linear regime of functional monovalency, and that binding led to endocytosis (Supplementary Figure 1). This conjugation scheme left a substantial fraction of the QD population unbound to EGF, which is non-consequential for these studies, as QD binding events were EGF-specific based on a competition assay and absence of bare QD binding to cell (Supplementary Figure 2).

We use compact alloyed QDs that we recently developed with hydrodynamic dimensions near 10 nm (Supplementary Figures 4–5)^23^, compared with 15–35 nm sizes for commercial variants, to be near the 8-nm spacing between adjacent EGF molecules in EGFR oligomers so as to avoid steric hindrance impacts on signaling^24^. Binding isotherms on MDA-MB-231 cells measured by flow cytometry showed nearly identical affinity for the QD-EGF conjugates (*K*_D_ = 3.1 ± 0.6 nM) compared to EGF conjugated to a single tetramethylrhodamine dye (dye-EGF; *K*_D_ = 3.2 ± 0.4 nM) (Fig. 2c), which has a similar binding affinity as unlabeled EGF^25^. Measured *K*_D_ values were in the range of those reported previously for EGF-EGFR binding on other cell types^26,27^. The similar affinity is logical as the *k*_on_ for EGF-EGFR binding is orders of magnitude smaller than that of a diffusion-controlled reaction^28^, and the diffusion coefficient of QD-EGF is just 4–5 times larger than that of dye-EGF. In addition, dye-EGF and QD-EGF conjugates resulted in similar number of fluorescent endosomes (Supplementary Figure 6), which is consistent with previous findings and indicates similar degrees of receptor activation^29^.

Compact QDs with fluorescence in the visible spectrum have similar intensities to cellular autofluorescence in epi-illumination mode (Fig. 2d–f), making absolute quantification in 3D impossible. Measurements using fluorescent dyes are much worse; single dye-EGF conjugates were eight times dimmer than mean cellular autofluoresence (Fig. 2e, f) and more than 10 times dimmer than QD605. We thus tuned the QD emission through ternary compositional alloying of the core to the near-infrared, beyond 700 nm, where autofluorescence is substantially attenuated (Fig. 2d), resulting in facile identification of individual QDs with 40-fold higher mean intensity than autofluorescence (Fig. 2f) for high-accuracy quantification with an area under receiver operating characteristic (AUROC) of 0.99 (Fig. 2g). Notably, in this spectral window, the dye intensity is still comparable in intensity to autofluorescence. This unique utility of QDs to emit with high intensity in the near-infrared for low-background cellular imaging adds to the growing value of these materials for applications such as deep-tissue imaging^30^.

Absolute quantification of EGF bound to a cell with a thickness of ~10 μm requires photostable labels because imaging the entire 3D cell volume requires repeated excitation to acquire sequential image planes at sufficient *z*-axis resolution. QDs are expected to outperform dyes for this application due to their exceptional photostability that exceeds that of dyes by orders of magnitude^31,32^. We labeled cells with a combination of dye-EGF and QD744-EGF and reconstructed 3D images from slices that were either acquired from the bottom to the top of a cell (Fig. 2h) or from the top to the bottom (Fig. 2i). The observed distribution of intensity for the dye-EGF conjugate was substantially different between the two acquisition processes, with photobleaching clearly apparent in the slices acquired at later times in both cases. In contrast, QD-EGF showed similar intensity distributions for both acquisition routines, highlighting the benefit of QD photostability.

### Fluorescence intensity quantization

**Fig. 3:**
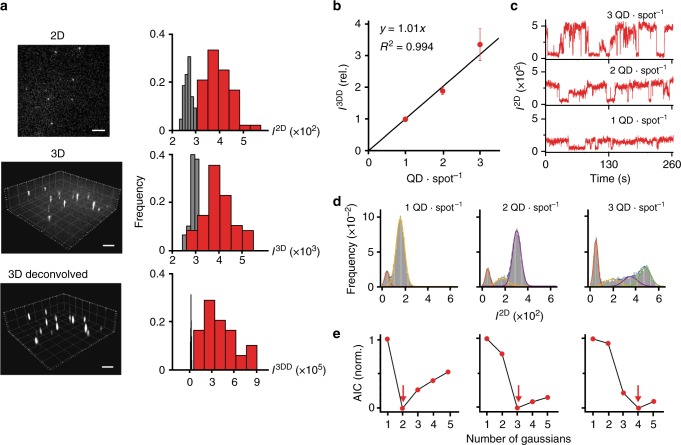
Intensity calibration for counting growth factors. **a** Representative images show QD744 imaged in two-dimensional (2D), three-dimensional (3D), and 3D after deconvolution. Histograms depict noise (gray) and quantum dot (QD) intensities (red). Voxel sizes are 3 × 3 for 2D and 3 × 3 × 11 for both 3D and deconvolved 3D. *N* = 48. Scale bar, 5 µm. **b** Intensity calibrated deconvolved 3D analysis of diffraction-limited spots containing 1, 2, or 3 QDs. Points indicate mean ± s.e.m. with *N* = 72, 216, and 27 spots for 1, 2, and 3 QD spot^−1^, respectively. The number of QDs per diffraction-limited spot is calculated from distributions of intensity from time traces based on the number of Gaussians fitted to brightness histograms. The number of QDs per spot is the number of fitted Gaussian minus 1 (noise)**. c** Examples of intensity time traces of 1, 2, and 3 QD spot^−1^. **d** Gaussian fitting for intensity histograms of 1, 2, and 3 QD spot^−1^ corresponding to examples shown in **c**. **e** Minima of Akaike information criteria (AIC) are indicated by red arrows, corresponding to the optimal number of Gaussians to fit each intensity histogram

While absolute quantification of QDs and fluorescent molecules on flat surfaces (e.g., coverslips, basolateral membranes, and microbes) with sparse labeling is well established^33^, counting in 3D presents unique challenges for intensity calibration due to autofluorescence, out-of-focus signals, and random single-QD blinking. Compared with 2D images, the intensity of a single near-infrared QD in 3D overlaps substantially with background (AUROC = 0.96), even in the absence of cells (Fig. 3a). Deconvolution reassigns out-of-focus light back to its point of origin to increase signal-to-noise ratio^34^, which we observe increases QD intensity significantly over background, with unity AUROC. Shown in Fig. 3b, deconvolved 3D intensities of single QDs verify that multiple QDs in a single diffraction-limited 3D spot can be accurately counted. This deconvolved intensity was independent of the distance across the thickness of a cell (Supplementary Figure 7). We analyzed 1, 2, and 3 QD spots, identified by their distinguishable 2D intensity time–trace distributions resulting from blinking, with example data shown in Fig. 3c–e. The quantized number of QDs contributing to each distribution was determined by fits validated by Akaike information criteria (AIC)^35^, with examples shown in Fig. 3e. This outcome is important because EGFR oligomers and coalesced receptors within endosomes will contain numerous EGFs within a diffraction-limited volume.

### EGF quantification

**Fig. 4:**
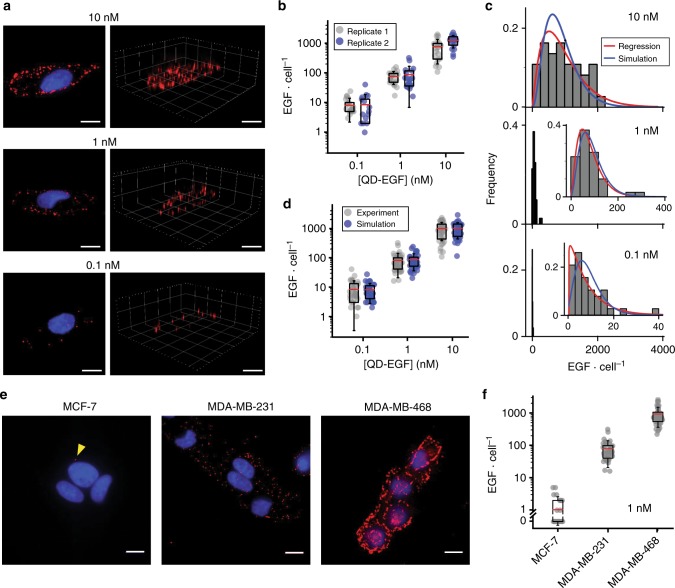
Counting and simulating growth factor binding. **a** Representative z-projections of maximum intensity (left) and three-dimensional (3D) images (right) of cells treated with indicated quantum dot-epidermal growth factor (QD-EGF) concentration for 5 min on ice. **b** Number of QD-EGF bound per cell at indicated QD-EGF concentrations, showing two independent replicates with *N* ≥ 17 cells for each condition. **c** Distributions show the number of EGF per cell measured experimentally at the indicated QD-EGF concentration. Maximum likelihood estimation regressions of gamma distributions are shown as red lines and simulation results are shown as blue lines. For regression, *p* = 0.79, 0.88, and 0.85 for 0.1, 1, and 10 nM QD-EGF concentrations, respectively. For simulation, *p* = 0.24, 0.49, and 0.67 for 0.1, 1, and 10 nM QD-EGF concentrations, respectively. All *p* values were calculated using *χ*^2^ tests. **d** EGF per cell is shown as experimental results (gray) and simulation results (blue) across different concentrations. Simulation results in **d** were obtained by sampling cells from the EGFR number gamma distribution (see Methods). **e** Representative z-projections of maximum intensity of breast cancer cell lines MCF-7, MDA-MB-231, and MDA-MB-468 in order of increasing EGFR expression. Cells were treated with 1 nM QD-EGF for 5 min on ice. Yellow arrow indicates a single QD-EGF bound to an MCF-7 cell. **f** Number of QD-EGF bound per cell for conditions in **e** with *N* ≥ 40 cells for each condition. QDs are shown in red and nuclei are blue. In **a**, **e**, QDs are shown in red and nuclei are blue; scale bars, 10 µm. In **b**, **d**, **f**, the box indicates 25/75th percentile; red lines are means; whiskers are s.d.

We used QDC-3DM to count the number of EGF molecules in individual MDA-MB-231 cells using a commonly applied temporal-pulse stimulation experiment^36^. Cells were exposed to QD-EGF conjugates across four log(10)-spaced concentrations for 5 min on ice, and unbound conjugates were washed away. Figure 4a shows example images of cells treated with 0.1, 1, and 10 nM concentrations of QD-EGF, across which counts per cell ranged from 0 to 4000, with mean values linearly correlated with concentration between 0.1 to 10 nM (Fig. 4b and Supplementary Figure 8a), and some binding saturation at 100 nM (Supplementary Figure 9). Importantly, mean stimulation numbers were reproducible between two independent experimental replicates (Fig. 4b and Supplementary Figure 8a) and EGF distributions fit well to gamma distributions (*p* ≥ 0.79 by *χ*^2^ test) (Fig. 4c, red regressions), insinuating a correlation with intrinsic distribution in receptor number^2,5,37,38^. We simulated the bound ligand distribution by applying a ligand–receptor kinetic binding model^12,28,39^ to known distributions of EGFR expression in MDA-MB-231 cells^37,38,40^, with ligand binding further distributed by a Poisson to simulate ligand binding probabilities per cell (see Methods). The experimental EGF binding distribution matched simulations well both at 37 °C (Supplementary Figure 10) and at 4 °C (Fig. 4c, d and Supplementary Figure 8b), with <13% deviation in mean stimulation magnitude between theory and experiment (Supplementary Table 1). Deviations between simulation and experiment were largest for the distribution width at 0.1 nM QD-EGF, for which the coefficient of variation was measured to be 95% but predicted to be 65%, suggesting strong merit in empirical measurements at low physiological stimulation levels, likely deriving from intrinsic noise effects such as local fluctuations in ligand and receptor concentrations^39,41^.

QDC-3DM also allows absolute counting of EGF binding events on cells with widely ranging EGFR expression. Figure 4e shows images of three human breast cancer cell lines after a pulse of 1 nM QD-EGF on ice. An increase in the number of EGF bound is evident as EGFR expression increases from low (MCF-7; ~10^4^ cell^−1^)^42,43^ to medium (MDA-MB-231; ~10^5^ cell^−1^)^42,43^ to high (MDA-MB-468; ~10^6^ cell^−1^)^42^. There were a mean of 1, 80, and 922 EGF per MCF-7, MDA-MB-231, and MDA-MB-468 cells, respectively (Fig. 4f), and a large fraction of MCF-7 cells were bound to 0 or 1 EGF bound (Fig. 4e, f), highlighting the necessity of single-molecule counting to assess absolute stimulation. Importantly, EGF binding was almost exclusive to EGFR, as EGF binding was reduced to <0.2% per cell after treatment with Cetuximab, which specifically blocks the EGFR binding site for EGF (Supplementary Figure 11)^44^.

### EGF spatial distribution

**Fig. 5:**
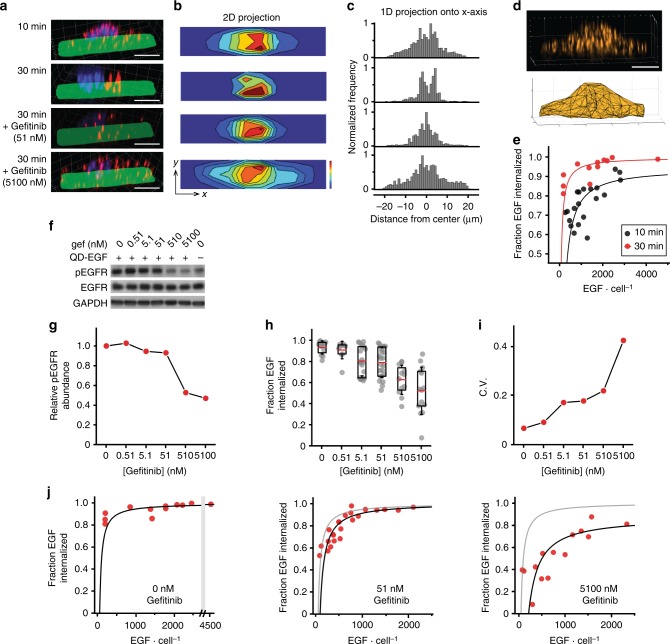
Single-cell epidermal growth factor (EGF) binding correlates with single-cell receptor translocation and drug response. **a** Representative three-dimensional (3D) images of MDA-MB-231 cells after stimulation with quantum dot-EGF (QD-EGF) in the absence or presence of EGFR inhibitor gefitinib. Times after the start of a stimulation pulse are indicated. QDs are shown in red, nuclei are blue, and Alexa Fluor 488-conjugated fibronectin micropatterns are green. **b** Two-dimensional (2D) *z*-projections on *xy* fibronectin micropattern planes and **c** one-dimensional (1D) projections on *x*-axes indicate the localization of single EGF averaged across cells. **d** Representative image of cell membrane measured through fluorescence imaging of fluorescently labeled receptors (top) and membrane reconstruction using alpha shapes (bottom). **e** Correlation between EGF number and fraction of EGF internalized in individual cells at 10 and 30 min after the start of a QD-EGF stimulation pulse. **f** Western blots and **g** relative pEGFR abundance in MDA-MB-231 whole-cell lysates immediately after stimulation with QD-EGF in the presence of indicated gefitinib concentrations. Uncropped western blots with molecular weight markers are shown in Supplementary Figure 16. **h** Fraction of EGF internalized in single cells at different gefitinib concentrations, 30 min after the start of a QD-EGF pulse. The box indicates 25/75th percentile; red lines are means; whiskers are s.d. **i** Coefficient of variation (CV) of the fraction of EGF internalized in **h**. **j** Number of EGF bound impacts the fraction of EGF internalized 10 min after the start of a QD-EGF pulse in the presence of gefitinib at 0, 51, and 5,100 nM concentration. The gray line shown in 51 nM (middle) and 5100 nM (right) gefitinib plots is the linear fit for 0 nM gefitinib condition (left). Data fits are shown in Supplementary Figure 14. *N* = 20 and 12 cells for 10 and 30 min after QD-EGF stimulation onset without gefitinib, respectively; *N* = 12, 10, 20, 23, 12, and 14 cells for 30 min after QD-EGF stimulation onset in the presence of gefitinib at 0, 0.51, 5.1, 51, 510, and 5100 nM concentrations, respectively. All stimulation pulses used 1 nM EGF-QD for 5 min. All scale bars indicate 10 µm

We demonstrate an example of how QDC-3DM provides empirical correlations between input stimulus and output signaling in single cells based on the localization of EGF, reflecting receptor internalization over time. As a prototypical receptor tyrosine kinase, EGFR undergoes phosphorylation on intracellular domains upon ligand binding, as well as internalization by endocytosis and intracellular trafficking, while signals propagate to downstream kinase cascades^45^. To correlate translocation across multiple cells, micro-contact printed hydrogel matrices were used to normalize adhesive footprints for cultured cells and ensure uniform distributions of size, shape, and organelle location^36,46^. Figure 5a shows representative images of individual cells at early (10 min) and late (30 min) stages after 5 min pulsed EGF stimulation, with EGF labeled in red, showing translocation from surface regions to perinuclear regions consistent with late endosomes or lysosomes over time. Example images also show inhibition of internalization by the EGFR-blocking drug gefitinib at two concentrations^47^. These 3D images of patterned cells can be reduced to 2D heat map averages across populations of cells (Fig. 5b) and 1D projection histograms (Fig. 5c) to depict the ensemble average of how receptor-ligand signaling events propagate spatially within cells. Notably EGFR was substantially redistributed across the cell with EGF treatment (Supplementary Figure 12).

### Stimulation magnitude correlation with signaling

With these cell imaging tools together, we can extract a wealth of single-cell signaling analytics that are both absolute in molecular number and spatially resolved across any cell across the stimulation distribution. EGF translocation metrics, in particular, are intrinsic signal propagation outputs of the analysis which can be readily correlated to the distance from the membrane using 3D membrane maps which we reconstruct as surfaces using alpha shapes^48^ derived from membrane labels in a separate QD channel (Fig. 5d and Supplementary Figure 13). We observed that in the absence of inhibitors, the number of internalized EGF after a 5 min pulse was linearly proportional to the number of EGF bound with a slope near 1 and *R*^2^ ≥ 0.99 (Supplementary Figure 14a). Trends are enhanced when plotted as fraction internalized in Fig. 5e, showing 10 and 30 min after stimulation. The *y*-axis spread in values indicates that the heterogeneity of internalization is greater at shorter time periods, while the differences in x-intercepts indicate the internalization rate. Between 10 and 30 min, the number of internalized EGF increases by ~120 independently of stimulation value between ~300 and ~2700. We also observed that the absolute number of QD-EGF per cell decreased from ~1300 to ~800 between 10 and 30 min after a 5 min pulse (Supplementary Figure 15a) likely due to dissociation of QD-EGF from the receptors after the pulse, an outcome that can be directly probed with QDC-3DM.

### Stimulation impact on pharmacological inhibition

Receptor internalization was attenuated by pharmacological EGFR inhibition in a dose-dependent manner that was coupled to EGF stimulation magnitude. Importantly, EGFR is a widely pursued proto-oncogene drug target, and blocking activation correlates both with inhibition of phosphorylation and translocation, which blocks downstream signals driving chemotaxis and proliferation^49^. Unfortunately, drugs targeting EGFR activation such as gefitinib have limited clinical efficacy in cancers such as triple-negative breast cancer, in which up to 76% of cases overexpress EGFR^49^. Western blot analysis of MDA-MB-231 cells exposed to 1 nM QD-EGF conjugates (1267 ± 788 EGF per cell) showed substantial receptor phosphorylation and a dose-dependent decrease in phosphorylation with increasing drug concentration (Fig. 5f–g and Supplementary Figure 16) with half-maximal inhibitory concentration (IC_50_) of 195 nM. Figure 5h shows the dose dependence of EGFR internalization fraction, exhibiting a population-averaged potency of inhibition (IC_50_ = 380 nM) similar to that of phosphorylation inhibition and slightly higher than the average equilibrium binding constant to the receptor *K*_D_ (51 nM)^50^. At the single-cell level, an increase in drug concentration led to higher variability in response of individual cells (Fig. 5i), with coefficient of variation monotonically increasing from 7.0 to 43% between 0 and 5.1 μM, an effect that has been widely reported for many classes of inhibitors^51^. Note that there was no significant difference in EGF binding for drug concentrations between 0 and 5.1 μM (Supplementary Figure 15b, c).

Figure 5j shows how cell-to-cell variability of EGFR inhibitor response derives substantially from the magnitude of EGF stimulation. At a drug concentration near the *K*_D_ (51 nM), EGF internalization remained similarly proportional to EGF bound across all cells, but with shifted internalization fraction that was equally diminished in magnitude across the population (see also Supplementary Figure 14b), suggesting uniform deactivation of membrane-localized EGFR. Moreover, these correlations demonstrated that excess stimulation was sufficient to overcome the biological effect of inhibition, with only 5% drug effect measured for 1500 EGF bound, compared with a 44% drug effect for 200 EGF bound. At 100-fold higher inhibitor concentration (5100 nM), internalization was further reduced, with 25% drug effect at 1500 EGF and 100% effect for 200 EGF bound. From these correlations it is apparent how stimulation can overcome signaling depletion thresholds imposed by inhibitors, and how heterogeneity arises from the proportionality between internalization and stimulation. By mapping the stimulation distribution (*x*-axes in Fig. 5j) to the internalization fraction slope, it can be seen that the low stimulation fraction, where slope is highest, has a dominant contribution to heterogeneity. The slope decreases for higher drug concentrations, so a greater percentage of the stimulation then contributes to the spread of drug effects, as the number of active receptors become depleted to yield a more stochastic population response.

## Discussion

Counting individual growth factors using QDC-3DM requires a combination of molecular probe properties that is uniquely provided by QDs. Tuning emission to the infrared while retaining efficient blue excitation eliminates the vast majority of cellular autofluorescence needed to boost signal-to-noise and increase the accuracy of single-molecule identification from 74 to 99% (Fig. 2g). In comparison, fluorescent dyes are too dim and do not provide photostability needed to withstand continuous excitation during volumetric image acquisition of 100–200 z-planes (Fig. 2h, i). QDs further provide a convenient means to internally calibrate spot intensities to discrete ligand numbers due to distinctive binary emission signatures of single QDs derived from on-and-off single-QD blinking (Fig. 3b, c). However, these intensities measured in 3D only correlate well with discrete QD numbers when 3D images are deconvolved to boost spot intensities and compensate for light from outside the focal plane (Fig. 3a).

Blinking can impact each QDC-3DM step depicted in Fig. 1b, so the analytical performance can depend on both the QD photophysical properties and the image acquisition conditions. The primary interference is that QDs may transition between an “on” and “off” state during the image acquisition time window, so measured intensities can be intermediate between the two states. For the first step of QD time–trace acquisition to identify single QDs, this intermediate intensity could lead to misidentification of single QDs by an automated algorithm, particularly for QDs in the population with short on-time probabilities. We apply stringent criteria^18^ so that single-QD exclusion is more common than inclusion of QD multiplets. QDs with higher on-time fractions may correlate with brighter QDs in the population^52^, which could propagate to a calibrated 3D QD intensity that is skewed toward higher values in QDC-3DM step 2. The 3D intensities also depend on blinking in step 3, as some fraction of QDs will remain off over some of the 3D slices, contributing to the 3D intensity distribution width in Fig. 3a, b. Importantly, while off-time probabilities are largely independent of image acquisition conditions and QD structure, on-time probabilities can deviate due to a number of variables, particularly excitation intensity and QD surface passivation^53,54^, so different integration times may yield different relative intensities of specific QDs across a population with a distribution of blinking kinetics. For this reason, we used QDs and conditions for which deviations are expected to be minimal, using a thick insulating shell (4.7 monolayer (ML) CdZnS), low laser power (photon flux <10 mW cm^−2^), and short exposure time (~100 ms). Further increasing the shell thickness would reduce the number of QDs with low on-time probabilities and truncated on-time kinetics^55,56^. We found that the structure applied here provides an excellent fluorescence intensity together with a balanced physical size, yielding, together with the polymeric coating and a nanoparticle that is 12.6 nm in hydrodynamic diameter, comparable to common biological macromolecules such as antibodies. An increase in shell size to further reduce blinking could be offset by using a smaller core but at the expense of a wider emission band^18^, or by using a thinner coating, which could destabilize conjugates or lead to nonspecific binding. Efforts are underway to further optimize both nanocrystals and coatings to yield still smaller, brighter probes^57^, and further exploit wavelengths deeper in the infrared where Stokes shift is further increased and autofluorescence is further reduced^30^.

Using QDC-3DM, it is now possible to directly measure biochemical input signals in single cells where inference from output metrics was only possible previously^8^. Single-cell studies in both prokaryotic and eukaryotic cells have shown that most protein expression number distributions are described by a gamma distribution^37,38,40^. Likewise, we observe that single-cell growth factor binding distributions to MDA-MB-231 cells fit gamma distributions across three orders of magnitude of concentration (Fig. 4c). From simulations, distribution widths derive primarily from receptor number distributions convolved with a lesser contribution from intrinsic noise of random binding. Importantly, the contribution from intrinsic noise becomes larger at lower ligand concentrations, insinuating that for experiments under such conditions that simulate relevant physiological tissue states^9^, stimulation magnitudes cannot be directly inferred from receptor numbers. Simulations matched the experimentally measured mean ligand number quite well, but experimental distributions were consistently wider by a small margin (Supplementary Table 1), likely deriving from a combination of uncertainty in kinetic rate constants and receptor number distributions, as well as distributions of receptor states involving oligomerization and inhomogeneous localization in membrane microdomains. Notably, autocrine stimulation of cells by secreted factors will be undetectable by this technique, so it is important to determine whether such contributions may confound the analysis of the biological system under study. While MDA-MB-231 cells do not secrete EGF, they do produce noncanonical ligands for EGFR, but at levels far lower than what would contribute during the brief pulsed experiments applied here^58^.

QDC-3DM allows empirical mapping between quantized single-cell stimulation and single-cell signaling, allowing extraction of single-cell signaling metrics that are otherwise unobtainable. Because localization and translocation are core contributors to signaling^59^, we used receptor internalization as an easily measured physical corollary of ligand-induced signal propagation downstream of receptor activation by phosphorylation. We find that the correlation between internalized EGF and the number of EGF bound shifts uniformly with time across the cell population (Fig. 5e and Supplementary Figure 14a). The stimulation distribution further modulates the response to the EGFR inhibitor gefitinib (Fig. 5j and Supplementary Figure 14b), diminishing drug effects at high EGF stimulation, and mediating heterogeneity at low EGF stimulation. These outcomes suggest that the concentration of growth factors in cell culture medium and local concentrations within tissue microenvironments will dictate drug–response sensitivity and heterogeneity based on how stimulation distributions map to sensitivity curves. These observations are most relevant to human cancers that develop diverse mechanisms to dysregulate EGFR signaling, including overexpression of receptors and overproduction of ligands, resulting in a resistance to signaling inhibition by targeted drugs^60–62^.

In conclusion, we developed a functional near-infrared QD suitable for single-molecule counting in autofluorescent cells, as well as a detailed methodology for absolute quantification of growth factors on single cells using 3D fluorescence microscopy. We applied this approach to count growth factors under physiologically relevant stimulation conditions spanning three log(10)-spaced stimulation magnitudes. As a microscopy-based assay, this technology is well suited for pairing with downstream analyses of signaling and phenotype through live-cell fluorescent protein imaging, immunofluorescence, fluorescence in situ hybridization, and high-content microfluidics, and can further be adapted to long-term tracking and steady-state stimulation experiments beyond the acute pulsed experiments used here. The combined capabilities of spatially registered signaling events through cellular micropatterning and highly multiplexed fluorescent color-coding using QDs can form the components of a toolbox for elucidation of signaling biology to connect individual molecular events to comprehensive cell response and population distributions. We expect that this toolbox can be applied to any peptide ligand and used broadly to provide a more comprehensive understanding of the origin of cell heterogeneity and drug effect variability.

## Methods

### Chemicals and reagents

Cadmium acetate hydrate (Cd(Ac)_2_·H_2_O, 99.99+%), mercury acetate (Hg(Ac)_2_, 99.999%), selenium dioxide (SeO_2_, ≥99.9%), selenium powder (Se, ~100 mesh, 99.99%), sulfur powder (S, 99.98%), octanethiol (OT, ≥98.5%), behenic acid (BAc, 99%), 1,2-hexadecanediol (HDD, 97%), tetramethylammonium hydroxide solution (TMAH, 25wt.% in methanol), *N*-methylformamide (NMF, 99%), *N*,*N*,*N*′,*N*′-tetramethylethylenediamine (TEMED, 99%), (3-aminopropyl)triethoxysilane (APTES, 99%), glutaraldehyde, sodium periodate (99%), and 2-azidoacetic acid (97%) were purchased from Sigma-Aldrich. Anhydrous cadmium chloride (CdCl_2_, 99.99%) and zinc acetate (Zn(CH_3_COO)_2_, 99.98%) were obtained from Alfa Aesar. 1-Octadecene (ODE, 90% tech.), oleylamine (OLA, 80–90% C18 content), oleic acid (OAc, 90% tech.), and hydrazine hydrate (55%) were purchased from Acros Organics. DBCO-sulfo-NHS ester was purchased from Click Chemistry Tools. Sodium bicarbonate and glycine were purchased from Thermo Fisher Scientific. Polydimethylsiloxane (PDMS) was purchased from Polysciences. Acrylamide and bisacrylamide were purchased from Bio-Rad. Glacial acetic acid (99.7%) was purchased from JT Baker. Solvents including chloroform, hexane, toluene, methanol, acetone, and diethyl ether were purchased from a variety of sources, including Acros Organics, Thermo Fisher Scientific, and Macron Fine Chemicals. All chemicals above were used as purchased.

Dulbecco’s modified Eagle’s Medium (DMEM), fetal bovine serum (FBS), Hank’s balanced salt solution (HBSS), and cell cultured grade bovine serum albumin (BSA) were purchased from VWR. SAv was purchased from ProSpec. Biotinylated EGF, dye-EGF, Hoechst, Alexa Fluor 488 NHS Ester, Alexa Fluor 647-conjugated goat anti-mouse antibodies, and goat serum were purchased from Thermo Fisher Scientific. Paraformaldehyde (PFA, 32% v/v in water) was purchase from Electron Microscopy Sciences. Dimethyl sulfoxide (DMSO), fibronectin from human plasma, Accutase cell detachment solution, and Tris hydrochloride (Tris-HCl, 1 M) were purchased from Sigma. Biotinylated DNA was prepared by Integrated DNA Technologies. MemBrite Fix 640/660 Cell Surface Staining Kit was purchased from Biotium. Phosphate-buffered saline (PBS) was purchased from Corning. His-tag protein A and Cetuximab were purchased from BioVision. Mouse monoclonal immunoglobulin G (IgG) antibody against human EGFR (EGFR.1 clone) was purchased from BD Biosciences. EGF and rabbit monoclonal IgG antibody against EGFR used in western blotting were purchased from Abcam. Mouse monoclonal IgG antibody against phosphorylated EGFR was purchased from R&D Systems. Horseradish peroxidase-conjugated antibodies against mouse and rabbit IgG were ordered from Jackson ImmunoReserach Laboratory. Gefitinib (>99%) was purchased from LC Laboratories. Western blotting reagents including Tris, sodium chloride (NaCl), ethylenediaminetetraacetic acid (EDTA), Triton X-100, sodium dodecyl sulfate (SDS), deoxycholate, sodium fluoride (NaF), sodium metavanadate (NaVO_3_), Tween-20, glycerol, bromophenol blue, and tris(2-carboxyethyl)phosphine (TCEP) were purchased from various sources including Sigma, Thermo Fisher Scientific, and Bio-Rad.

### Synthesis of quantum dots

QD cores composed of core/shell CdSe/Cd_*y*_Zn_1− y_S (QD565 and QD605) or Hg_*x*_Cd_1 −x_Se/Cd_*y*_Zn_1 − *y*_S (QD744) were synthesized in-house^18^ and coated with the multidentate polymer polyacrylamindo(histamine-*co*-triethyleneglycol) (P-IM) or polyacrylamindo(histamine-*co*-triethyleneglycol-*co*-azido-triethylene-glycol) (P-IM-N_3_). These polymers yield particles with compact hydrodynamic diameter (7–12 nm) with nearly monomeric size distributions by gel permeation chromatography (>98%). The QDs are functionalized with azides for P-IM-N_3_ coatings^23^. The QD565 and QD605 cores were reported in our previously published manuscript^23^ while QD744 was synthesized using the process described below.

CdSe QDs with 3.2 nm diameter were prepared by a heat-up synthesis method and then exchanged with mercury to yield an alloyed Hg_*x*_Cd_1− x_Se core. Cd(BAc)_2_ (0.2 mmol), SeO_2_ (0.2 mmol), HDD (0.2 mmol), and ODE (4 mL) were mixed in a 50-mL round bottom flask and dried under vacuum at ~100 °C for 1 h. The temperature was raised to 230 °C at a rate of ~20 °C min^−1^ under nitrogen gas and maintained at 230 °C for 15 min. The solution was then cooled to ~110 °C by removing the heating mantle, and the QDs were purified by dilution with chloroform (10 mL) containing OAc (1 mL) and OLA (0.6 mL), and precipitation with a mixed solvent of methanol (15 mL) and acetone (15 mL). The QDs were redispersed in hexane and extracted twice with methanol followed by precipitation with excess methanol. Finally, the QDs were dispersed in a chloroform solution containing OAc and OLA (20 mL, chloroform:OAc:OLA = 20:1:1 by volume). Mercury exchange was initiated by injecting a mercury stock solution (Hg(Ac)_2_ in OLA, 0.1 M) into the CdSe solution at room temperature with vigorous stirring. The ratio between total Cd atoms in the CdSe QDs and the injected Hg cations was 1:2. The reaction was allowed to continue for 5 min and then quenched by adding excess OT (~20 eq. to Hg^2+^). Aliquots (0.2 mL) were collected before mercury addition and 3 min after OT addition, and absorption spectra were measured to analyze spectral shifts and extinction coefficient changes. The resulting Hg_*x*_Cd_1 −*x*_Se QDs were purified by precipitation with a methanol/acetone mixture (50% v/v, ~30 mL) containing OAc (~0.2 mL) and OLA (~0.2 mL). The QDs were redispersed in chloroform (~15 mL) containing OAc (~0.2 mL) and OLA (~0.2 mL) and precipitated again by the addition of methanol/acetone (~30 mL). This dissolution–precipitation process was repeated three times to completely remove unreacted Hg(Ac)_2_ and any reaction byproducts. Finally, the pure Hg_*x*_Cd_1 − *x*_Se QDs with band edge absorption at ~640 nm were dispersed in hexane.

A Cd_*x*_Zn_1 −*x*_S shell was deposited epitaxially over the Hg_*x*_Cd_1 −*x*_Se QD cores^23^. Purified QDs in hexane (~100nmol) were transferred to a 50-mL round bottom flask and the solvent was evaporated under nitrogen flow at 40–50 °C. The dried QDs were immediately redispersed in a mixed solvent of ODE (2 mL) and OLA (1 mL) containing sulfur precursor (S in ODE, 0.1 M) for the first 0.8 MLs of shell. The temperature was raised to ~120 °C under nitrogen and maintained at this temperature for 10 min. Then Cd_*x*_Zn_1 −*x*_ precursor (*x*:1 − *x* mixture of Cd and Zn precursors, Cd(Ac)_2_ and Zn(Ac)_2_ in OLA, 0.1 M) in an equivalent mole quantity to the previous sulfur precursor was added dropwise while raising the temperature to ~130 °C. The reaction was allowed to proceed for 10 min at this temperature. This 0.8-ML shell growth cycle was repeated while controlling the composition (*x*) and raising the reaction temperature. Detailed reaction parameters for QD744 are summarized in Table 1 for a nanocrystal with band edge absorption wavelength of 702 nm, peak fluorescence emission of 744 nm with a full width at half maximum of 75 nm. Electron microscopy characterization as well as absorption and fluorescence emission spectra are shown in Supplementary Figure 5.

**Table 1:** Shell growth conditions for QD744

Shell thickness	Shell	Reaction temperature (°C)
2.4 ML, in three increments of 0.8 ML	CdS	40–170
0.8 ML	Cd_0.5_Zn_0.5_S	180–190
0.8 ML	Cd_0.25_Zn_0.75_S	200
0.8 ML	ZnS	200

### Polymer coating of QDs

QD565, QD605, and QD744 (~18 µM) were coated with either P-IM or P-IM-N_3_ using a two-step process^23^. First, QDs in hexane (0.5 mL) were purified by precipitation by mixing with chloroform (1.5 mL) and acetone (4 mL). The QD pellet was redispersed in hexane (4 mL) and extracted three times with methanol. The purified QDs (~2.5 µM, 3 mL) were mixed with NMF (2 mL) and TMAH solution (195 µL) in a glass vial and vigorously stirred for 1 h until all QDs transferred to the NMF phase. The transparent QD dispersion in NMF (1 nmol, ~280 µL) was diluted with DMSO (750 µL) in a glass vial equipped with a magnetic stir bar. P-IM or P-IM-N_3_ dissolved in DMSO (11.3 mg mL^−1^, ~159 µL) was added dropwise to the QDs while stirring. This mixture was then bubbled with nitrogen for 2 min and stirred for 2 h at 110 °C. The solution was then cooled to room temperature and the QDs were precipitated with the addition of ether (5 mL) and chloroform (2 mL). The QD pellet was dispersed in sodium borate buffer (50 mM, pH 8.5). Excess polymer was removed by filtration with a 50 kDa molecular weight cutoff (MWCO) Amicon ultra-centrifugal filter (Millipore), and finally dispersed in sodium borate buffer. Homogeneity and hydrodynamic size were analyzed through gel permeation chromatography, shown in Supplementary Figure 4a.

### Conjugation of QDs to EGF

P-IM-N_3_-coated QD565, QD605, and QD744 were conjugated to DBCO-functionalized SAv by click-mediated triazole formation, and then conjugated to EGF through a single N-terminal biotin. QDs were conjugated to SAv using the following protocol^23^. SAv (180 µL, 0.5 mg mL^−1^) was first mixed with a 5-fold molar excess of DBCO-sulfo-NHS ester (1.6 µL, 5 mM in DMSO) and incubated on ice for 2 h. The reaction was quenched by dilution with a Tris-HCl (9 µL, 1 M) solution. Unreacted DBCO-sulfo-NHS ester was removed by filtration using a 0.5 mL Amicon centrifuge filter with 30 kDa MWCO. It was previously verified that these reaction conditions yielded nearly 1:1 conjugates between QDs and SAv^23^, further confirmed by nearly complete shifts of agarose gel electrophoresis bands of the QDs after SAv conjugation; bands further shifted after addition of biotinylated 90-mer single-stranded DNA, shown in Supplementary Figure 4b. The DNA sequence was 5′-Biotin/(T)_68_ TAG CCA GTG TAT CGC AAT GAC G-3′. DBCO-SAv was then mixed with P-IM-N_3_-coated QDs at a 1:1 molar ratio (0.5 µM) at 4 °C for 12 h. Then, a 50-fold molar excess of 2-azidoacetic acid was added and unreacted reagents were removed by filtration with a 0.5 mL Amicon centrifuge filter with 100 kDa MWCO. QD-SAv was then conjugated to EGF-biotin by mixing EGF-biotin with QD-SAv at specific ratios to a final QD concentration of 0.2 µM in PBS at 4 °C for 4 h. Gel electrophoresis with a hybrid polyacrylamide (PA)-agarose gel (2% PA and 0.5% agarose) was used to characterize the conjugates^23,63^. To ensure that the conjugation between the QD-SAv and biotin-EGF was functionally monovalent, we varied the ratio of biotin-EGF:QD-SAv and observed that a dose response in cells followed a linear trend with increasing conjugation ratio until saturation (Fig. 2b). Thus by choosing a biotin-EGF:QD-SAv of 0.33:1, well within the linear regime, we could ensure that the QD-EGF complex was largely monovalent. We have also verified that these QD-EGF conjugates are highly specific and functional (Supplementary Figures 1–2).

### Conjugation of QDs to IgG

P-IM-coated QD605 was conjugated to a monoclonal IgG antibody against EGFR (EGFR.1 clone) through a protein A linker. Protein A contained a single his-tag, allowing rapid, efficient, and functional conjugation to QDs with P-IM coatings by metal chelation of the QD surface^23^. First, the QDs were mixed with a 4-fold molar excess of his-tag protein A in PBS at a QD concentration of 1 µM at room temperature for 2 h. Then, anti-EGFR IgG was added at a molar ratio of 4:1 IgG:QD in PBS to reach a QD concentration of 0.8 µM. The mixture was incubated at room temperature for 3 h and then stored at 4 °C until use. Thirty minutes prior to use, the IgG conjugates were diluted in serum-free, phenol red-free DMEM supplemented with 0.8% BSA.

### Fibronectin labeling

Alexa Fluor 488-labeled fibronectin was prepared by mixing Alexa Fluor 488 NHS ester and fibronectin from human plasma (1 mg mL^−1^, 1 mL) at a 10:1 molar ratio in 0.1 M sodium bicarbonate buffer (pH 8.3) at room temperature for 1 h in the dark. Unreacted dye was quenched by the addition of glycine (20 mM), followed by 10 min of incubation and purification using a MiniTrap Sephadex G-25 column (GE Healthcare) with PBS mobile phase. After purification, there was a mean 4.5 Alexa Fluor 488 molecules per fibronectin based on ultraviolet–visible absorption spectrophotometry. Immediately before use, Alexa Fluor488-labeled fibronectin (25 µg mL^−1^, 1 mL) was oxidized with sodium periodate (3.5 mg mL^−1^) for 45 min at room temperature to form ketones. The oxidized protein solution was then filtered through a 0.2 µm syringe filter.

### Hydrogel substrate preparation

PA hydrogels were fabricated on glass coverslips (18 mm, Thermo Fisher Scientific)^64,65^. First, coverslips were washed with ethanol and deionized water. Each coverslip was placed in a well of a 12-well plate and amine-functionalized with 1 mL APTES (0.5% v/v in deionized water) at room temperature for 3 min. Coverslips were then washed three times with deionized water, followed by 1 mL glutaraldehyde (0.5% v/v in deionized water) at room temperature for 30 min to generate aldehydes. A stock PA solution prepared by mixing acrylamide (25 mL, 20%) and bisacrylamide (4.9 mL, 2%) was passed through a 0.2 µm cutoff filter and degassed by bubbling with nitrogen. For each sample, ammonium persulfate (0.1%) and TEMED (0.1%) were added to the PA solution to initiate cross-linking. The PA solution (20 µL) was then sandwiched between the functionalized glass coverslip and glass slide with hydrophobic surface for 20 min. The hydrogel-coated glass coverslips were then detached from the glass slide and placed in wells of a 12-well plate. The hydrogel surfaces were treated with hydrazine hydrate for 2 h, rinsed with 5% glacial acetic acid for 1 h, and finally incubated in deionized water overnight. Immediately before use, PA hydrogels were dried at room temperature for 1.5 h and sterilized under ultraviolet light for 15 min.

### Micro-contact printing of fibronectin on hydrogels

Microislands of fluorescent fibronectin were deposited by stamping onto PA hydrogels as 500µm^2^ rectangles with specific aspect ratios (5, 1.5, and 1). A PDMS stamp was fabricated by polymerization on a patterned master of photoresist (SU-8, MicroChem) coated on a silicon wafer by photolithography. PDMS stamps were cleaned with ethanol and sterile water immediately before use. Oxidized Alexa Fluor 488-labeled fibronectin (25 µg mL^−1^, 150 µL) was then added to the top of the patterned PDMS stamp and allowed to adsorb for 30 min. Excess fibronectin solution was quickly removed under nitrogen air stream and the fibronectin-coated PDMS surface was immediately transferred to the dried PA hydrogel by stamping. The fibronectin printed PA hydrogel was then submerged in PBS in a 12-well plate and was ready for cell seeding.

### Isolated QDs on glass coverslips

P-IM-N_3_-coated QD744 in PBS (1 nM) were spin coated (2500 rpm, 30 s) onto #1.5 glass coverslips that were cleaned with ethanol, methanol, and acetone.

### Unpatterned cells without QD treatment

MCF-7 cells (ATCC, HTB-22), MDA-MB-231 cells (ATCC, HTB-26), or MDA-MB-468 cells (ATCC, HTB-132) (50,000 cells mL^−1^, 0.5 mL) were cultured on Lab-Tek II eight-well chamber slides (Nunc) in phenol red-free DMEM supplemented with 10% FBS. After 8 h, the cells were starved overnight in serum-free, phenol red-free DMEM containing 0.8% BSA. The cells were then fixed with 4% PFA in PBS on ice for 15 min, washed three times with ice-cold PBS, and permeabilized with methanol on ice for 6 min. Cells were then stained with 1 µg mL^−1^ Hoechst at room temperature for 10 min and washed three times with PBS.

### Unpatterned cells treated with QD-EGF or dye-EGF

Samples were prepared similarly to unpatterned cells without QD treatment with the following changes: after overnight starvation, the medium was removed and replaced with ice-cold serum-free, phenol red-free DMEM supplemented with 0.8% BSA containing different concentrations of QD-EGF and/or dye-EGF. Cells were incubated on ice for 5 or 10 min, and then washed three times with ice-cold PBS and then fixed, permeabilized, and stained with Hoechst as described above.

### Unpatterned cells for EGF internalization assay

Samples were prepared similarly to unpatterned cells without QD treatment with the following changes: after overnight starvation, the medium was removed and replaced with pre-warmed serum-free, phenol red-free DMEM supplemented with 0.8% BSA containing different concentrations of QD-EGF, dye-EGF, or QD-SAv. Cells were incubated at 37 °C for 5 min, and then washed three times with pre-warmed serum-free, phenol red-free DMEM supplemented with 0.8% BSA. Cells were further incubated at 37 °C for 25 min, then fixed, permeabilized, and stained with Hoechst as described above.

### Patterned cells treated with QD-EGF

To visualize the spatial localization of QD-EGF across multiple cells, cells were shaped to specific geometries by growth on islands using the micro-contact printing methodology described above. MDA-MB-231 cells (30,000 cells mL^−1^, 1 mL) in phenol red-free DMEM supplemented with 10% FBS were seeded into each well of a 12-well plate containing coverslips with fibronectin patterned PA hydrogels. After 2.5 h, cells were starved in serum-free, phenol red-free DMEM supplemented with 0.8% BSA for 5 h. Cells were then treated with QD744-EGF (1 nM) in the same medium for 5 min. Cells were then washed three times with serum-free medium and maintained for specific time periods in serum-free medium. The cells were washed three times with ice-cold serum-free medium and incubated with QD605-IgG (20 nM) on ice for 6 min. Cell were washed three times with ice-cold PBS, fixed, and stained with Hoechst as described in Protocol 2.

### Patterned cells treated with QD-EGF and gefitinib

Samples were prepared similarly to patterned cells treated with EGF-QD with the following changes: after starvation, cells were treated with different concentrations of gefitinib as indicated for 40 min in serum-free DMEM. The medium was removed and replaced with ice-cold serum-free, phenol red-free DMEM supplemented with 0.8% BSA containing QD744-EGF and the same concentration of gefitinib for 5 min. Cells were then washed three times with serum-free medium and maintained in serum-free medium with the same concentration of gefitinib for the indicated time. The cells were then treated with the QD605-IgG membrane stain according to Protocol 5, and the remainder of the protocol was followed.

### Patterned cells treated with QD-EGF and Cetuximab

Samples were prepared similarly to patterned cells treated with EGF-QD with the following changes: after starvation, cells were treated with Cetuximab (20 nM) as indicated for 1.5 h in serum-free DMEM. The medium was removed and replaced with pre-warmed serum-free, phenol red-free DMEM supplemented with 0.8% BSA containing 1 nM QD744-EGF and the same concentration of Cetuximab for 5 min. Cells were then washed three times with serum-free medium and maintained in serum-free medium with the same concentration of Cetuximab for the indicated time. Cells were then then fixed, permeabilized, and stained with Hoechst as described above.

### Patterned cells with membrane stain

Samples were prepared similarly to patterned cells treated with EGF-QD with the following changes: after starvation, cells were stained with the MemBrite Fix Cell Surface Staining Kits following the manufacturer’s protocol. Briefly, cells were treated with pre-staining solution in HBSS at 37 °C for 5 min. Cells were then treated with staining solution diluted in HBSS (1:1000 dilution) at 37 °C for 5 min. The cells were washed three times with ice-cold serum-free medium, and treated with the QD605-IgG membrane stain according to Protocol 5. The remainder of the protocol was followed.

### Patterned cells with EGFR stain

Samples were prepared similarly to patterned cells treated with EGF-QD with the following changes: after starvation, cells were washed three times with PBS, fixed, and permeabilized as described above. Cells were then blocked with 1% BSA in PBS at room temperature for 15 min and stained with mouse anti-EGFR antibody (1 μg mL^−1^) in 1% BSA at 4 °C overnight. After incubation, cells were washed three times with PBS and blocked with 1% BSA and 2% goat serum in PBS at room temperature for 15 min. Cells were then stained with Alexa Fluor 647-conjugated goat anti-mouse secondary antibody (1:300 stock dilution) and Hoechst (1 μg mL^−1^) at room temperature for 1 h.

### Western blot

MDA-MB-231 cells (300,000 cells) were seeded in each well of a 6-well plate for 72 h in DMEM supplemented with 10% FBS. Cells were then starved in serum-free DMEM supplemented with 0.8% BSA for 5 h. Serum-starved cells were then treated with gefitinib at the indicated concentrations for 40 min in serum-free DMEM containing with 0.8% BSA. Cells were then stimulated with QD744-EGF (1 nM) in the presence of different concentrations of gefitinib for 5 min and washed three times with ice-cold Tris-buffered saline (TBS; 50 mM Tris, 150 mM NaCl, pH 7.5). Cells were lysed by treatment with radioimmunoprecipitation assay buffer (50 mM Tris, 150 mM NaCl, 2 mM EDTA, 1% Triton X-100, 0.1% SDS, 0.5% deoxycholate) supplemented with Halt Protease Inhibitor Cocktail (Thermo Fisher Scientific) and phosphatase inhibitors (50 mM NaF, 1 mM NaVO_3_) on ice for 15 min. Cell lysates were collected after centrifugation for 15 min at 14,000*g* at 4 °C; a small fraction was aliquoted for protein concentration measurement using the bicinchoninic acid assay. Protein concentrations for each sample were adjusted to ~0.9 mg mL^−1^. Cell lysates were then mixed with 5× sample buffer (1 M Tris, pH 9, 10 g SDS, 12.5 mL glycerol, 100 µL 0.5 M EDTA, 50 mg bromophenol blue, 100 mM TCEP) to a final concentration of 1×, heated at 75 °C for 20 min, aliquoted, and stored at −80 °C until use.

Samples were loaded into wells of an SDS-polyacrylamide gel; electrophoresis was performed, and gels were transferred to a polyvinylidene difluoride membrane (Immubilon-P membrane, Millipore). The membrane was washed three times with deionized water followed by Tween-20 (0.1%) in TBS for 5 min each. The membrane was then blocked with 5% milk and 0.1% Tween-20 in TBS for 1 h. The membrane was treated overnight at 4 °C with a solution of primary antibodies in 1% milk and 0.1% Tween-20 in TBS. Primary antibodies used were rabbit anti-EGFR (1:500 dilution), mouse anti-human pEGFR (1:250 dilution), and rabbit anti-glyceraldehyde 3-phosphate dehydrogenase (GAPDH) (1:1000 dilution; Cell Signaling). Membranes were washed with 1% milk and 0.1% Tween-20 in TBS five times before incubation with horseradish peroxidase-conjugated secondary antibodies (anti-mouse or anti-rabbit, 1:5000 dilution) for 1 h. Membranes were again washed five times with 1% milk and 0.1% Tween-20 in TBS, and one time with 0.1% Tween-20 in TBS before bands were developed by enhanced chemifluorescence substrate (ECL, Thermo Fisher Scientific) and imaged on autoradiography film (Denville Scientific). Images were analyzed using ImageJ software (National Institutes of Health). The band intensities for pEGFR and EGFR were divided by that of GAPDH; then, the band intensity of pEGFR/GAPDH was divided by EGFR/GAPDH. The intensities were normalized to sample treated with 1 nM QD-EGF without gefitinib to calculate the ratio of pEGFR to total EGFR under the different experimental conditions.

### Flow cytometry

MDA-MB-231 cells were seeded in a T-75 cell culture flask in DMEM supplemented with 10% FBS and cultured until 90% confluence. Cells were washed once with PBS and treated with 5 mL Accutase at room temperature until fully detached from the surface. Accutase was removed by centrifugation for 5 min at 200*g* and cells were washed once with ice-cold PBS containing 0.5% BSA and resuspended in the same medium at 3 × 10^6^ cells mL^−1^. Cell suspensions were then mixed in equal volume (25 μL) with ice-cold solutions of QD-EGF (0.06–120 nM; EGF:QD = 0.33) or dye-EGF (0.02–40 nM). Control samples to measure nonspecific binding were prepared identically but with 2 μM unlabeled EGF. The cells were incubated at 4 °C for 4 h with rocking, washed three times with ice-cold PBS containing 0.5% BSA, and resuspended in PBS.

Fluorescence intensities of cells were measured with 488 nm laser excitation, a 685 LP dichroic mirror, and a 695/40 nm BP emission filter for QD-EGF, or 561 nm laser excitation and 582/15 nm BP emission filter for dye-EGF. Single cells were selected using a forward scatter width gate and a minimum of 10,000 single cells were measured for each condition. The percent of maximum EGF bound for each condition, *P*(*c*), was calculated using the following equation:

2 $$P(c) = \frac{{\overline {I_{c,{\mathrm{tot}}}} - \overline {I_{c,{\mathrm{ns}}}} }}{{\overline {I_{c_{{\mathrm{max}}},{\mathrm{tot}}}} - \overline {I_{c_{{\mathrm{max}}},{\mathrm{ns}}}} }} \times 100,$$

where $\overline {I_{c,{\mathrm{tot}}}}$ and $\overline {I_{c,{\mathrm{ns}}}}$ are the mean fluorescence intensities of cells treated with *c* concentration of QD-EGF or dye-EGF in the absence and presence of unlabeled EGF, respectively, and $\overline {I_{c_{{\mathrm{max}}},{\mathrm{tot}}}}$ and $I_{c_{{\mathrm{max}}},{\mathrm{ns}}}$ are the mean fluorescence intensities of cells treated with the maximum concentration (*c*_max_) of QD-EGF or dye-EGF in the absence and presence of unlabeled EGF, respectively. The dissociation constant, *K*_D_, was calculated based by fitting the QD-EGF or dye-EGF binding curve to the following equation using Prism (Graphpad Software):

3 $$P(c) = \frac{{B_{{\mathrm{max}}} \cdot c}}{{K_{\mathrm{D}} + c}},$$

where *B*_max_ is the maximum percent of specific binding.

### 2D and 3D microscopy

Fluorescence microscopy of isolated QDs and cells was performed using wide-field illumination on a Zeiss Axio Observer Z1 inverted microscope with a ×100 1.45NA alpha Plan-Fluar oil immersion objective, 100 W halogen lamp illumination, 488 nm/100 mW OPSL laser, and 561 nm/40 mW diode laser units. Images were acquired using a Photometrics eXcelon Evolve 512 EMCCD camera through the Zeiss ZEN software. Excitation light was filtered using Semrock and Zeiss filters (G 365, BP 470 nm/40 nm, BP 482/18, BP 561/14 nm). Emission signals were filtered using Semrock bandpass filters (445/50, 525/50, 562/40, 600/37, and 732/68 nm). Brightfield images were acquired using transmitted-light illumination (12 V, 100 W Halogen lamp) with DIC prism III/0.55.

### Cellular autofluoresence spectrum measurement

Cellular autofluorescence spectra were acquired with 488 nm excitation using two different instruments. For wavelengths between 530 and 727 nm, a Zeiss 710 confocal scanner Azio Observer Z1 inverted confocal microscope with a ×63 1.4 NA oil immersion objective and a tunable Mai-Tai Ti-Sapphire laser (Spectra Physics) with 488 nm laser excitation was used. Intensities were acquired using a QUASAR 34 channel spectral detector with 9.7 nm wavelength increments. For wavelengths above 727 nm, measurements were performed using the Zeiss Axio Observer Z1 inverted microscope described above using bandpass filters with one redundant wavelength to that for the confocal scanner to allow normalization of the data between the two instruments.

Individual cells from samples prepared using Protocol 2 were imaged to collect autofluorescence intensity measurements at a specific emission wavelength, *I*_AF_(*λ*_em_), normalized to the detector sensitivity using the equation below:

4 $$I_{{\mathrm{AF}}}(\lambda _{{\mathrm{em}}}) = \frac{{\overline {I_{{\mathrm{px}},{\mathrm{cell}}}(\lambda _{{\mathrm{em}}})} - \overline {I_{{\mathrm{px}},{\mathrm{b}}}(\lambda _{{\mathrm{em}}})} }}{{\mathop {\int }\nolimits_{\lambda _1}^{\lambda _2} {\mathrm{\Phi }}(\lambda ){\mathrm{d}}\lambda }},$$

where $\overline {I_{{\mathrm{px}},{\mathrm{cell}}}(\lambda _{\mathrm{em}})}$ is the mean pixel intensity on a cell at wavelength *λ*_em_, $\overline {I_{{\mathrm{px}},{\mathrm{b}}}(\lambda _{{\mathrm{em}}})}$ is the mean pixel intensity of background (non-cell regions) at wavelength *λ*_em_, $\mathop {\int }\nolimits_{\lambda _1}^{\lambda _2} {\mathrm{\Phi }}(\lambda ){\mathrm{d}}\lambda$ is the integrated quantum efficiency of the camera spanning the spectral channel bandwidth centered at wavelength *λ*_em_, and *λ*_1_ and *λ*_2_ are the lower and upper cutoff of the emission bandwidth. Autofluorescence at each wavelength was normalized by dividing by *I*_AF_ (562nm).

### Autofluorescence and single fluorophore intensities

Unpatterned cell samples were prepared as described above and stained with EGF conjugates of three different QDs emitting at 565, 605, and 744 nm or with a dye. The cells were then imaged at three emission wavelengths (562, 600, and 732 nm) for QDs under otherwise identical conditions and instrument settings or imaged with 561 nm laser excitation and 600 nm emission for the dye. Single QDs/dye were identified using methods^18^ in which videos of QD/dye spots were saved as TIFF stacks and imported into Matlab for QD/dye spot detection and single-QD/dye identification. QD/dye spot centroids (*x*_0_, *y*_0_) were obtained from images using the detection/estimation/deflation algorithm from the multiple-target tracing (MTT) algorithm of Sergé et al.^66^. Centroid locations were rounded to the closest integral pixel values, ([*x*_0_],[*y*_0_]), and an intensity histogram of a 3 × 3 pixel array centered at this position for the video was then fit to a sum of two functions, a Gaussian background (mean [*μ*_1_], standard deviation [*σ*_1_], and area [*a*_1_]) and a skewed Gaussian QD/dye signal (mean [*μ*_2_], standard deviation [*σ*_2_], area [*a*_2_], and skew factor [*r*]). Curve fits that satisfied previous criteria to distinguish single-QD/dye photophysical dynamics were used to identify single QDs/dyes, for which the intensity, *I*_QD/dye_(*λ*_em_), was determined as:

5 $$I_{{\mathrm{QD}}/{\mathrm{dye}}}\left( {\lambda _{{\mathrm{em}}}} \right) = \frac{{\mu _2 - \mu _1}}{{{\mathrm{\Phi }}(\lambda _{{\mathrm{em}}})}},$$

where Φ(*λ*_em_) is the quantum efficiency of the camera at wavelength *λ*_em_. Autofluorescence at a specific wavelength was calculated on the cell area for which there were no QDs, using the following equation:

6 $$I_{{\mathrm{AF}}}(\lambda _{{\mathrm{em}}}) = \frac{{\mathop {\sum }\nolimits_{x = [x_0] - 1}^{[x_0] + 1} \mathop {\sum }\nolimits_{y = [y_0] - 1}^{[y_0] + 1} I(x,y,\lambda _{{\mathrm{em}}}) - \overline {I_{3 \times 3,{\mathrm{b}}}(\lambda _{{\mathrm{em}}})} }}{{{\mathrm{\Phi }}(\lambda _{{\mathrm{em}}})}},$$

where *I*(*x*, *y*, *λ*_em_) is the intensity for pixel (*x*,*y*), $\overline {I_{3 \times 3,{\mathrm{b}}}(\lambda _{{\mathrm{em}}})}$ is the mean 3 × 3 pixel intensity sum of background regions, and ([*x*_0_], [*y*_0_]) is centroid of each 3 × 3 pixel array of autofluorescence.

### Deconvolution

3D volumetric stacks (250 nm z-spacing, 80–200 images) of QDs were deconvolved using AutoQuantX3 (Media Cybernetics). All stacks were deconvolved using the following settings: fixed point spread function (PSF), 60 iterations, and noise level low as recommended by Media Cybernetics. PSF images were experimentally acquired using fluorescent TetraSpeck microspheres (0.1 μm diameter; Thermo Fisher Scientific), and calculated using the PSF image processing tool in Zeiss ZEN software.

### Isolated QD intensity calibration

Two stacks of images of isolated QDs on glass coverslips were were collected in wide-field excitation mode: a time stack at a single z-focal plane (4000 images; 100 ms exposure time) and a 3D volumetric stack (250 nm z-spacing, 80 images; 100 ms exposure time). 3D z-stacks were deconvolved using AutoQuantX3. Using custom Matlab codes, the deconvolved 3D intensity of each spot $( {I_{{\mathrm{spot}}}^{3{\mathrm{DD}}}})$ was then calculated as the integrated intensity of a 3 × 3 × 11 voxel centered at the centroid position according to the following equation:

7 $$I_{{\mathrm{spot}}}^{3{\mathrm{DD}}} = \mathop {\sum }\limits_{x = [x_0] - 1}^{[x_0] + 1} \mathop {\sum }\limits_{y = [y_0] - 1}^{[y_0] + 1} \mathop {\sum }\limits_{z = [z_0] - 5}^{[z_0] + 5} I(x,y,z) - \overline {I_{3\times3\times11,{\mathrm{b}}}},$$

where [*x*_0_], [*y*_0_], and [*z*_0_] are the centroid positions rounded to the nearest pixel integer, *I*(*x*, *y*, *z*) is the intensity of a single pixel, and ${\overline {I_{3\times3\times11,{\mathrm{b}}}}}$ is the mean 3 × 3 × 11 voxel intensity sum of background region. Using the same 2D spot ([*x*_0_], [*y*_0_]) centroid positions, 3 × 3 time-course intensities $( {I_{{\mathrm{spot}}}^{2{\mathrm{D}}}})$ were calculated according to the following equation:

8 $$I_{{\mathrm{spot}}}^{2{\mathrm{D}}}(t) = \mathop {\sum }\limits_{x = [x_0] - 1}^{[x_0] + 1} \mathop {\sum }\limits_{y = [y_0] - 1}^{[y_0] + 1} I(x,y,t).$$

Using Matlab, all intensities for a spot were binned into a histogram composed of 100 bins. The intensity histogram was fitted using least square estimate to a Gaussian mixture model with 2–5 Gaussians, for which one was the background noise function corresponding to the off-state of QD blinking. To maximize the accuracy in fitting, we imposed the following fitting criteria: (1) correlation coefficient greater than or equal to 0.98 between the fit and data, (2) each Gaussian area contributes at least 8% the total area, (3) maximum 75% overlap between any two Gaussians, and (4) maximum 20% difference in area between each Gaussian and its corresponding data region. For each spot, the number of Gaussians that yields the minimum AIC value was identified as optimal. AIC was calculated according the following equation:

9 $${\mathrm{AIC}} = n_{{\mathrm{bin}}}{\mathrm{ln}}\left( {\frac{{\mathrm{RSS}}}{{n_{{\mathrm{bin}}}}}} \right) + 2(3n_{{\mathrm{Gauss}}} - 1),$$

where *n*_bin_ is the number of bins used to construct the intensity histogram, RSS is the residual sum of squares, and *n*_Gauss_ is the number of Gaussians used to fit the intensity histogram.

### QDC-3DM methodology

Two stacks of images of the QDs were collected in wide-field excitation mode: a time-stack at a single z-focal plane (600 images; 50 ms exposure time) and a 3D volumetric stack (250 nm z-spacing, 100–200 images; 50 ms exposure time). 3D z-stacks were deconvolved using AutoQuantX3. Deconvolved 3D images were then imported into Imaris (Bitplane) which has an automatic 3D detection algorithm (surface mode) to determine the centroid positions (*x*_0_, *y*_0_, *z*_0_) and intensity $( {I_{{\mathrm{spot}}}^{3{\mathrm{DD}}}})$ of spots with a range of sizes. These spot data, the time-stack images, and the deconvolved 3D images were imported into Matlab and a custom script was used to calculate the number of QD-EGF per cell.

[1] *Single-QD identification*: Spot positions (*x*_0_, *y*_0_, *z*_0_) were rounded to the nearest integer pixel values, ([*x*_0_], [*y*_0_], [*z*_0_]), and time-course intensities of the corresponding 2D spots, $I_{{\mathrm{spot}}}^{2{\mathrm{D}}}(t)$, were summed over a 3 × 3 voxel centered about the centroid positions ([*x*_0_], [*y*_0_]) at each time point using equation 8. Temporal intensities $I_{{\mathrm{spot}}}^{2{\mathrm{D}}}(t)$ for each spot were binned into histograms and fit to a sum of two functions, a Gaussian background and skewed Gaussian signal. Single QDs were identified from istribution fits that satisfy previous criteria to distinguish single-QD photophysical dynamics^18^.

[2] *Single-QD intensity calibration*: Deconvolved 3D spot intensities $( {I_{{\mathrm{spot}}}^{3{\mathrm{DD}}}})$ for which spots correspond to single QDs $( {I_{{\mathrm{spot}}}^{3{\mathrm{DD}}} = I_{1{\mathrm{QD}}}^{3{\mathrm{DD}}}})$ were averaged to calculate the mean single-QD intensity,

10 $$\overline {I_{1{\mathrm{QD}}}^{3{\mathrm{DD}}}} = \frac{1}{n}\mathop {\sum }\limits_{i = 1}^n I_{1{\mathrm{QD}}_i}^{3{\mathrm{DD}}},$$

where *n* is the number of QDs identified as single.

[3] *Spot intensity calibration*: The number of QDs within each deconvolved 3D spot, *N*_QD,spot_, for images collected under the same conditions and experimental set was then calculated as:

11 $$N_{{\mathrm{QD}},{\mathrm{spot}}} = I_{{\mathrm{spot}}}^{3{\mathrm{DD}}} \cdot \left( {\overline {I_{1{\mathrm{QD}}}^{3{\mathrm{DD}}}} } \right)^{ - 1}$$

For any 3D field of view, such as a single cell (*N*_QD,cell_) containing *m* spots, the total number of QDs can be calculated as the sum of QDs in each spot as:

12 $$N_{{\mathrm{QD}},\,{\mathrm{cell}}} = \mathop {\sum }\limits_{i = 1}^m N_{{\mathrm{QD}},\,{\mathrm{spot}}_i}.$$

### Internalization fraction calculation

Cells membranes were mapped using 3D images of QD605-IgG membrane stains using the Matlab alphaShape function by importing ([*x*], [*y*], [*z*]) coordinates of QD605-IgG with an alpha radius of 50. Spatial coordinates for QD605-IgG spots were obtained using the MTT detection/estimation/deflation algorithm^66^ for each 2D image of a 3D z-stack spanning the entire cell thickness. For spots detected in the same ([*x*], [*y*]) positions across adjacent z-planes, [*z*] values were averaged. Nucleus ([*x*_nuc_], [*y*_nuc_], [*z*_nuc_]) coordinates were determined using Imaris.

In Matlab, a vector was constructed connecting the nucleus and surface through each QD744-EGF spot centroid position ([*x*_QD_], [*y*_QD_], [*z*_QD_]) derived from the above deconvolved 3D images, with surface intersection coordinates ([*x*_surf_], [*y*_surf_], [*z*_surf_]). An EGF spot was identified as internalized if it satisfied the following condition of relative distance from the surface:

13 $$\left[ {\frac{{\left( {x_{{\mathrm{QD}}} - x_{{\mathrm{nuc}}}} \right)^2 + \left( {y_{{\mathrm{QD}}} - y_{{\mathrm{nuc}}}} \right)^2 + \left( {z_{{\mathrm{QD}}} - z_{{\mathrm{nuc}}}} \right)^2}}{{\left( {x_{{\mathrm{surf}}} - x_{{\mathrm{nuc}}}} \right)^2 + \left( {y_{{\mathrm{surf}}} - y_{{\mathrm{nuc}}}} \right)^2 + \left( {z_{{\mathrm{surf}}} - z_{{\mathrm{nuc}}}} \right)^2}}} \right]^{1/2} \le 0.8.$$

The fraction of EGF internalized (*f*) was then calculated using the following equation for a cell in which there are *n* spots internalized.

14 $$f = \frac{1}{{N_{{\mathrm{QD}},{\mathrm{cell}}}}}\mathop{\sum }\limits_{i = 1}^n N_{{\mathrm{QD}},{\mathrm{spot}}_i}.$$

### Membrane stain analysis

To evaluate the accuracy of the QD membrane stain, cells were co-stained with MemBrite according to Protocol 7. Volumetric images of membranes were collected using a Zeiss 710 confocal scanner Azio Observer Z1 inverted microscope with ×63 1.4 NA oil immersion objective with 250 nm z-spacing and 640/660 nm excitation/emission bands. The cell membranes at each z-plane of the confocal images were then manually segmented to serve as the membrane standard to calculate the accuracy of membrane maps obtained from QD605-IgG membrane stains and alpha shape analysis from epifluorescence images as described above  for calculating the internalization fraction. Differences in distances between the two cell membrane maps were calculated for each pixel of the membrane obtained via confocal and plotted in 3D using Matlab.

### 2D and 1D projections of EGF localization

Cells grown on micro-contact printed surfaces have the same adhesion shapes, which can be observed using Alex Fluor 488-labeled fibronectin. The fluorescent adhesion patterns were aligned using a custom Matlab code. The EGF locations were transformed similarly and projected either onto a 2D surface or 1D line.

### EGF-binding simulation

EGF-EGFR binding kinetics on a population of cells with heterogeneous EGFR expression was modeled using a Matlab code. The EGF-EGFR kinetic model involves three processes: association, dissociation, and internalization. Three differential equations were used to solve for the concentration of free receptor [EGFR](*t*), ligand–receptor complexes [EGF|EGFR](*t*), and internalized complexes [EGF|EGFR]_int_(*t*):

15 $$\frac{{\mathrm{d}}}{{{\mathrm{d}}t}}\left[ {{\mathrm{EGFR}}} \right]\left( t \right) = - k_{{\mathrm{on}}} \cdot \left[ {{\mathrm{EGF}}} \right]\left( t \right) \cdot \left[ {{\mathrm{EGFR}}} \right]\left( t \right) + k_{{\mathrm{off}}} \cdot \left[ {{\mathrm{EGF}}|{\mathrm{EGFR}}} \right](t),$$

16 $$\frac{{\mathrm{d}}}{{{\mathrm{d}}t}}\left[ {{\mathrm{EGF}}|{\mathrm{EGFR}}} \right]\left( t \right) = k_{{\mathrm{on}}} \cdot \left[ {\mathrm{EGF}} \right]\left( t \right) \cdot \left[ {\mathrm{EGFR}} \right]\left( t \right) - (k_{{\mathrm{off}}} + k_{{\mathrm{int}}}) \cdot \left[ {{\mathrm{EGF}}|{\mathrm{EGFR}}} \right](t),$$

17 $$\frac{{\mathrm{d}}}{{{\mathrm{d}}t}}\left[ {{\mathrm{EGF}}|{\mathrm{EGFR}}} \right]_{{\mathrm{int}}}\left( t \right) = k_{{\mathrm{int}}} \cdot \left[ {{\mathrm{EGF}}|{\mathrm{EGFR}}} \right](t),$$

where *k*_on_, *k*_off_, and *k*_int_ are kinetic rate constants for ligand–receptor association, ligand–receptor dissociation, and ligand–receptor internalization, respectively, provided in Tables 2 and 3.

**Table 2:** EGF-EGFR kinetic rate parameters at 37 °C

Constant	Value	Reaction	Reference
*k*_on_	1.03 × 10^6^ M^−1^ s^−1^	EGF + EGFR → EGF|EGFR	French et al.^28^
*k*_off_	5.67 × 10^−3^ s^−1^	EGF|EGFR → EGF + EGFR	Lauffenburger et al.^39^
*k*_int_	5.00 × 10^−4^ s^−1^	EGF|EGFR → EGF|EGFR_int_	Lauffenburger et al.^39^

**Table 3:** EGF-EGFR kinetic rate parameters at 4 °C

Constant	Value	Reaction	Reference
*k*_on_	1.03 × 10^4^ M^−1^ s^−1^	EGF + EGFR → EGF|EGFR	Approximation^a^
*k*_off_	2.84 × 10^−4^ s^−1^	EGF|EGFR → EGF + EGFR	Approximation^a^
*k*_int_	0 s^−1^	EGF|EGFR → EGF|EGFR_int_	Negligible at 4 °C^28,39^

^a^We assume that at 4 °C, *k*_on_ is 100× slower and *k*_off_ is 20× slower than at 37 °C^67,68^

Because experiments were performed in a large medium volume (*V*_cell_ ~16.7 nL extracellular volume per cell compared to ~1.7 pL intracellular volume for ~15 µm spherical cells), EGF concentration is approximately constant and equal to the initial value [EGF]_0_, which was 0.03, 0.3, 3, or 30 nM, corresponding to 0.1, 1, 10, or 100 nM of QD with QD:EGF = 3:1.

18 $$\frac{{\mathrm{d}}}{{{\mathrm{d}}t}}\left[ {{\mathrm{EGF}}} \right]\left( t \right) = 0;\left[ {{\mathrm{EGF}}} \right]\left( t \right) = \left[ {{\mathrm{EGF}}} \right]_{\mathrm{0}}.$$

The discrete steady-state population distribution of active EGFR copy number per cell (*N*_R_) is approximated as a gamma distribution^37,38,40^, for which:

19 $$p\left( {N_{\mathrm{R}}} \right) = \frac{{N_{\mathrm{R}}^{a - 1}e^{ - N_{\mathrm{R}}/b}}}{{\Gamma (a)b^a}}.$$

Here *Γ* is the gamma function, *a* is the inverse of noise $( {\overline {N_{\mathrm{R}}} ^2 \cdot \sigma ^{ - 2}})$ that defines the distribution shape, and *b* is the Fano factor $( {\sigma ^2 \cdot \overline {N_{\mathrm{R}}} ^{ - 1}})$ that defines the scale, or translation burst size. $\overline {N_{\mathrm{R}}}$ and *σ* are the mean and standard deviation of the protein number distribution, respectively. The average number of active receptors per cell is $\overline {N_{\mathrm{R}}} = 100,000\,{\mathrm{cell}}^{ - 1}$ based on the average EGFR number per MDA-MB-231 cell (200,000), of which ~50% are on the membrane^42,69^. Based on previous quantification of EGFR on MDA-MB-231 cells by flow cytometry using antibody fragments, we use *a* = 3.34^70^.

The rate equations were then solved for

20 $$N_{{\mathrm{EGF}}}(N_{\mathrm{R}}) = \left( {\left[ {{\mathrm{EGF}}|{\mathrm{EGFR}}} \right] + \left[ {{\mathrm{EGF}}|{\mathrm{EGFR}}} \right]_{{\mathrm{int}}}} \right) \cdot V_{{\mathrm{cell}}} \cdot N_{\mathrm{A}},$$

where *N*_A_ is Avagadro’s number. *N*_EGF_ is solved for each discrete *N*_R_ to yield the average number of EGF ligands for each discrete cell, $\overline {N_{{\mathrm{EGF}},{\mathrm{R}}}}$. Each average is then spread by a Poisson distribution to account for intrinsic noise^39^ as

21 $$p\left( x \right) = {\mathrm{e}}^{ - \bar x}\frac{{\bar x^x}}{{x!}},$$

where $\bar x = \overline {N_{{\mathrm{EGF}},{\mathrm{R}}}}$ and *x* = *N*_EGF,R_. Then, each *p*(*x*) = *p*(*N*_EGF,R_) is scaled by *p*(*N*_R_) and summed across *N*_R_ to generate the *N*_EGF_ distribution.

For Fig. 4c, the complete cell population was simulated. For Fig. 4d, individual cells were sampled from the *N*_R_ distribution in the same number as those in the experimental data, and then used to calculate the number of EGF bound by sampling the Poisson distribution spread of kinetic binding. The statistical difference between the distribution of *N*_EGF_ between experiment and simulation was calculated using the Mann–Whitney *U* test.

### Instrumentation

Cell and QD imaging was performed using a Zeiss Axio Observer Z1 inverted microscope for wide-field illumination in the Smith Lab, or a Zeiss 710 confocal scanner Azio Observer Z1 inverted microscope in the Carl R. Woese Institute for Genomic Biology core facility at the University of Illinois. Gel electrophoresis for QDs and QD conjugates was performed using an EPS-300X system (C.B.S. Scientific company Inc.). Gel images were collected using a Bio-Rad Molecular Imager Gel Doc XR system. Gel electrophoresis for western blot was performed using a Bio-Rad mini Protean tetra cell. Western blotting was carried out using a Bio-Rad Criterion Blotter and films were imaged using a Konica SRX-101A film processor. Flow cytometry data were acquired using a BD Biosciences LSR Fortessa Cytometry Analyzer equipped with 488 and 561 nm lasers in the Roy J. Carver Biotechnology Center at the University of Illinois. Absorption spectra of QDs were acquired using an Agilent Cary 5000 UV–Vis–NIR spectrometer. All measurements were carried out within the dynamic range of the instrument (absorbance < 4) in the entire spectral range. Fluorescence spectra of QDs using 491 nm excitation were acquired using a Horiba NanoLog spectrofluorometer. Raw fluorescence signal was adjusted for the wavelength-dependent detector sensitivity and excitation power fluctuations. Electron microscopy images were acquired using a JOEL 2010 LaB6 high-resolution microscope in the Frederick Seitz Materials Research Laboratory Central Research Facilities at the University of Illinois. Hydrodynamic sizes of QDs were measured via an ӒKTApurifier UPC10 (GE Healthcare) with a Superose™ 6 10/300GL column (GE Healthcare), controlled using the UNICORN 5.31 Workstation software. Photolithography was performed using a Karl Suss MJB3 Mask Aligner in the Micro and Nanotechnology laboratory at the University of Illinois.

### Statistical information

Except where otherwise noted, values are reported as mean ± standard deviation (s.d.). Statistical significance analyses were calculated using two-tailed Mann–Whitney test in Origin Pro 9.1. A statistically significant value was denoted with an asterisk (*) for *p* < 0.05. *χ*^2^ goodness-of-fit tests were performed using a built-in function in Matlab.

### Code availability

All codes used in this study are available from the corresponding author upon reasonable request.

## Supplementary information

Supplementary Information

Reporting Summary

## Data Availability

The data that support the findings of this study are available from the corresponding author upon reasonable request.
