# Supplementary material for: Counting growth factors in single cells with infrared quantum dots to measure discrete stimulation distributions
Source: Nat Commun. 2019 Feb 22;10:909. doi: 10.1038/s41467-019-08754-5 (PMC6385258; doi:10.1038/s41467-019-08754-5)
Supplement: Supplementary file 1 — Supplementary Information [file 41467_2019_8754_MOESM1_ESM.pdf]

Supplementary Information

Counting Growth Factors in Single Cells with Infrared Quantum Dots to Measure Discrete Stimulation Distributions

Le et al.

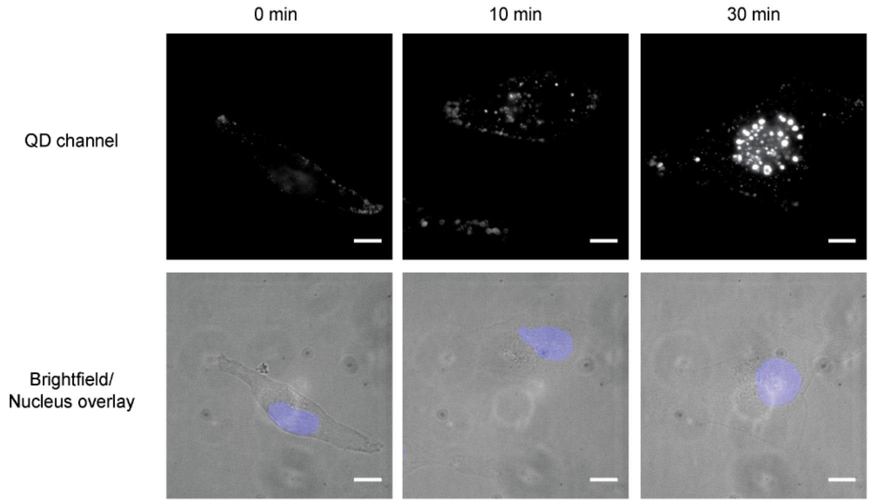

Supplementary Figure 1. **Biological functionality of QD-EGF**. MDA-MB-231 were treated with QD605-SAv-biotin-EGF (0.5 nM) at 37ºC for the indicated time to verify increasing internalization over time. Cells indicated as 0 min were treated on ice. Scale bar, 10 μm.

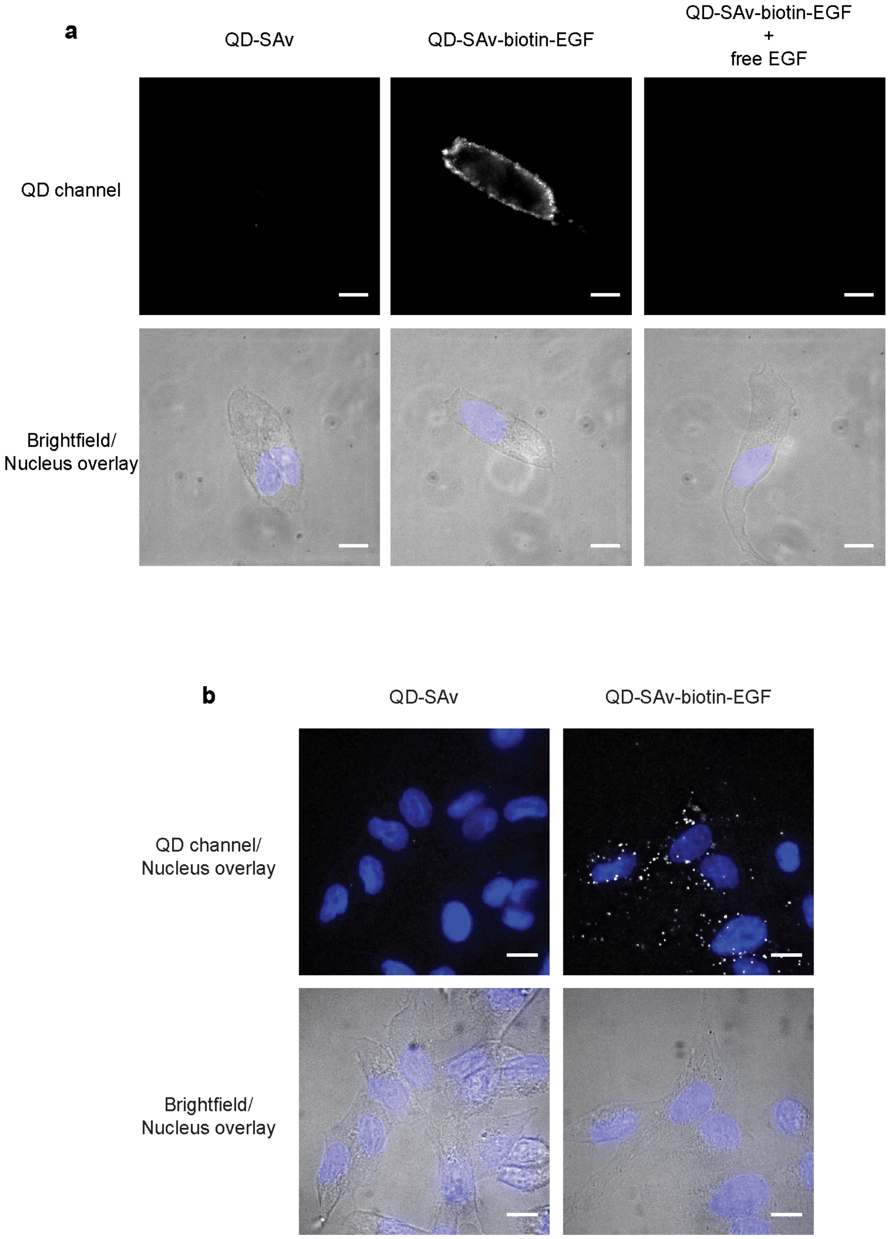

Supplementary Figure 2. **Binding specificity of QD-EGF**. **(a)** MDA-MB-231 were treated with QD605-SAv (1 nM) or QD605-SAv-biotin-EGF (1 nM; EGF:QD = 3:1) on ice for 10 min in the presence or absence of free EGF (160 nM) to block receptor binding. **(b)** MDA-MB-231 were treated with QD744-SAv (1 nM) or QD744-SAv-biotin-EGF (1 nM; EGF:QD = 0.33:1) for 5 min at 37ºC, washed 3 times with serum-free DMEM to remove excess QD, and further incubated at 37ºC for 25 min. Scale bar, 10 µm.

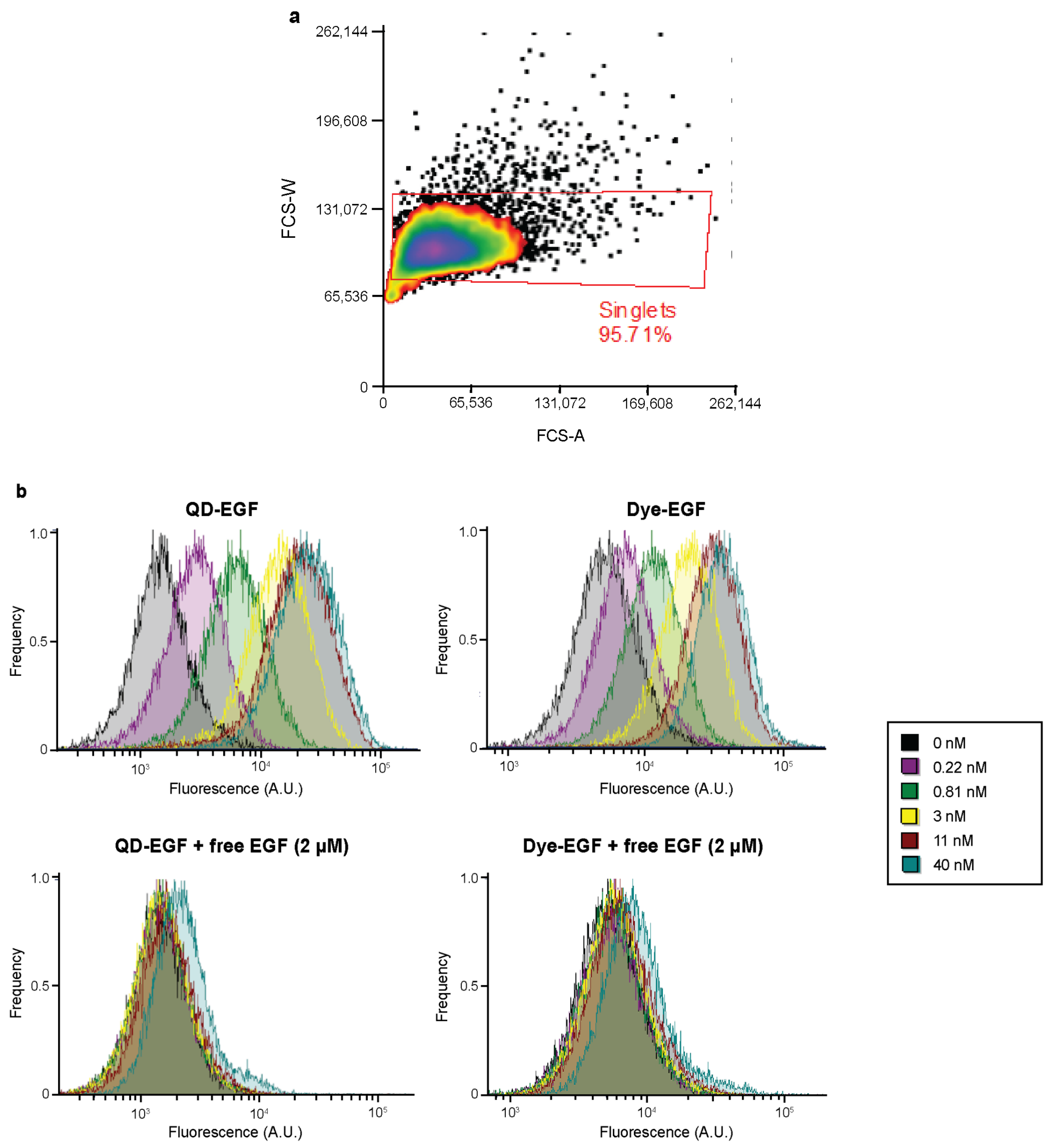

Supplementary Figure 3. **Representative flow cytometry data corresponding to Fig. 2c.** MDA-MB-231 were treated with indicated concentrations of QD-EGF (EGF:QD = 0.33:1) or dye-EGF at 4ºC for 4 hr in the presence or absence of free EGF (2 μM) to block receptor binding**. (a)** Representative gating used to isolate single cells. **(b)** Flow cytometry data of cells gated using strategy in (a).

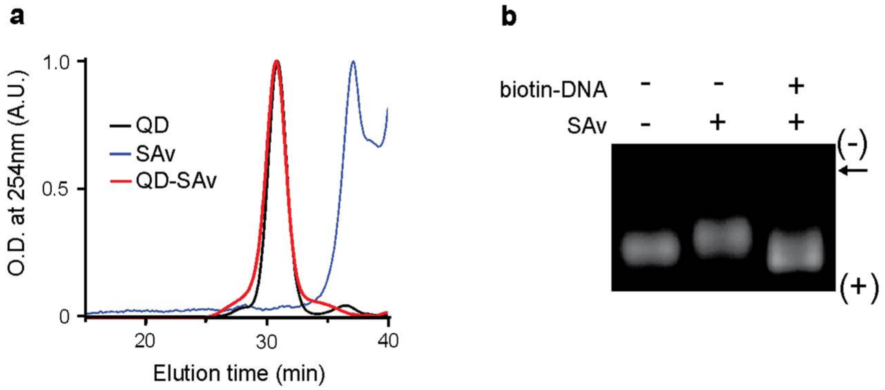

Supplementary Figure 4. **Hydrodynamic and functional characterization of aqueous QD744. (a)** Gel permeation chromatograms of QD744 coated with P-IM-N_3, QD744-SAv conjugates, and SAv alone**.** Globular protein size standards indicate that QD744 and QD744-SAv are homogeneous and have hydrodynamic diameters of 12.4 nm and 12.6 nm, respectively. The small degree of increase in hydrodynamic size with SAv conjugation is consistent with our recent studies of compact QD-SAv conjugates measured by fluorescence correlation spectroscopy, and is likely due to partial burial of SAv within the flexible QD coating.[1] **(b)** Agarose gel electrophoresis confirms complete conjugation between QD and SAv, and that QD-SAv can bind to biotin-DNA based on band shifts. Band signal derives from QD fluoresecence. QD-SAv (lane 2) migrates a shorter distance than QD (lane 1) due to the larger size of QD-SAv. The disappearance of the free QD band in lane 2 indicates complete conjugation between QD and SAv. QD-SAv-biotin-DNA (lane 3) migrates a longer distance than QD-SAv due to strong negative charge, indicating QD-SAv binding to biotin-DNA.

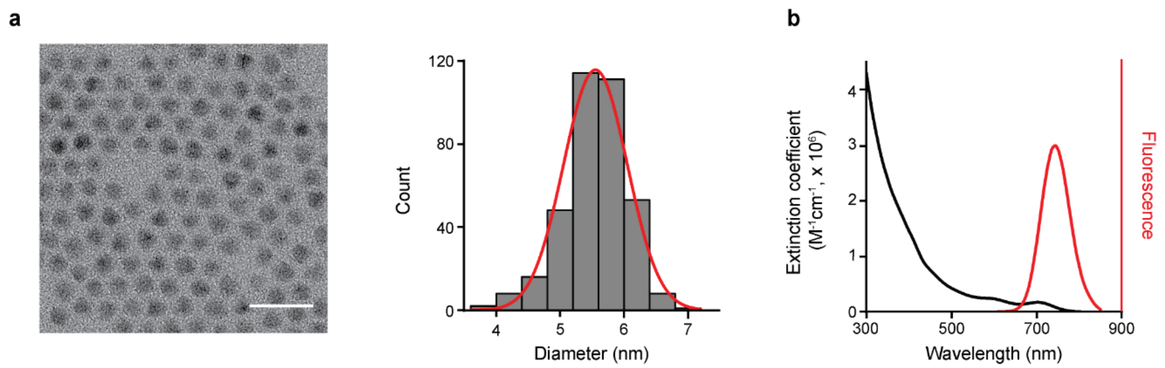

Supplementary Figure 5. **Structural and optical characterization of QD744. (a)** Representative transmission electron micrograph and size histogram of QD744 deposited from hexane. QD744 has a mean diameter of 5.55 ± 0.50 nm. Scale bar, 20 nm. **(b)** Extinction coefficient spectrum (black) and fluorescence emission spectrum (red) of QD744.

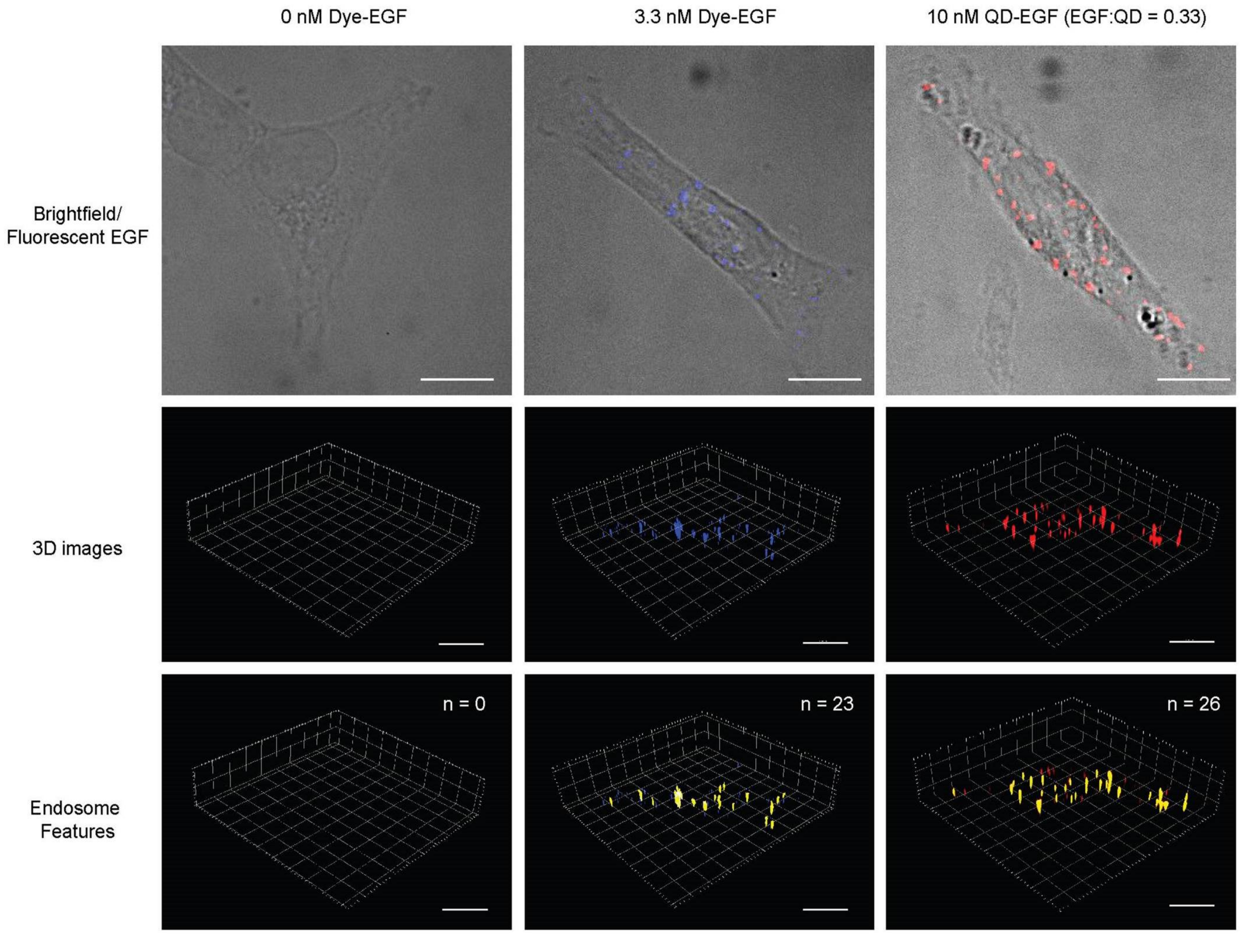

Supplementary Figure 6. **Endosome analysis of dye-EGF and QD-EGF.** MDA-MB-231 cells were treated with QD-EGF (EGF:QD = 0.33:1) or dye-EGF at 37ºC for 5 min, washed 3 times with serum-free DMEM to remove excess EGF, and incubated at 37ºC for an additional 5 min. Untreated cells were used to threshold autofluorescence. Imaris software was used to identify endosome features, defined as fluorescence spots brighter than autofluorescence and larger than 0.15 μm^3. The endosome threshold 0.15 μm^3 (diameter = 0.66 μm) was chosen as it is within the size range of endosomes[2] and ~3 times larger than the diffraction limit (~0.25 μm), allowing differentiation between single EGF spots and endosome containing multiple EGF. Endosome numbers are indicated by *n*. Scale bar, 10 μm.

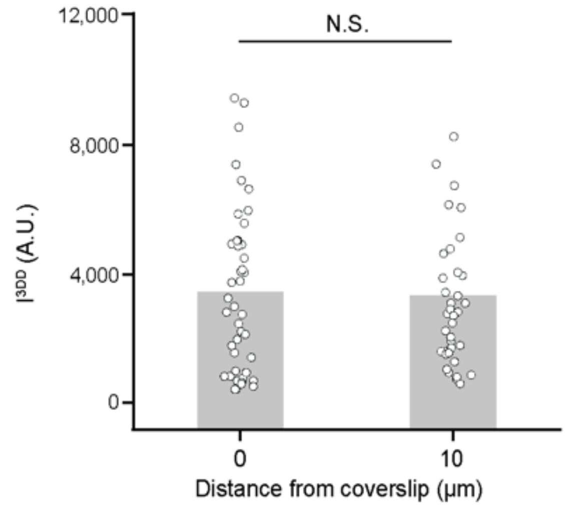

Supplementary Figure 7. **Impact of spatial location on deconvolved 3D intensity of single QDs.** MDA-MB-231 cells were treated with QD-EGF (1 nM) and single QDs on the cell bottom (0 μm from the coverslip) and top (~10 μm from the coverslip) were analyzed for 3D intensity after deconvolution. *N* = 43 and 37 QDs for 0 μm and 10 μm from the coverslip, respectively. N.S. indicates not significant ($p > 0.05$).

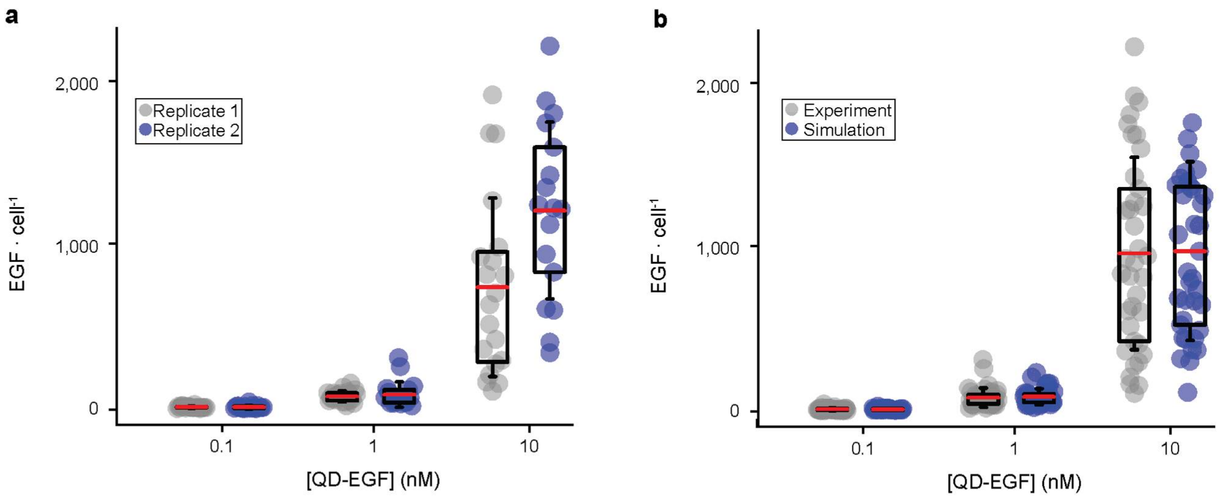

Supplementary Figure 8. **QD-EGF number per cell in semi-log scale corresponding to data in Fig. 4b and 4d. (a)** Number of QD-EGF bound per cell at indicated QD-EGF concentrations, showing two independent replicates with $N \geq 17$ cells for each condition. (**b)** Distributions show the number of EGF per cell measured experimentally at the indicated QD-EGF concentration. The box indicates 25/75th percentile; red lines are means; whiskers are s.d.

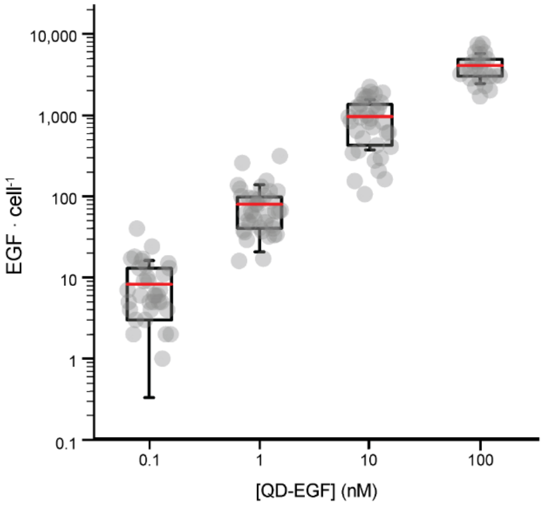

Supplementary Figure 9. **Distribution of QD-EGF number per cell at indicated QD-EGF concentrations**. *N* = 38, 40, 37, and 25 cells for 0.1, 1, 10, and 100 nM QD-EGF, respectively. The box indicates the 25/75th percentile; red lines indicate mean value; whiskers are s.d.

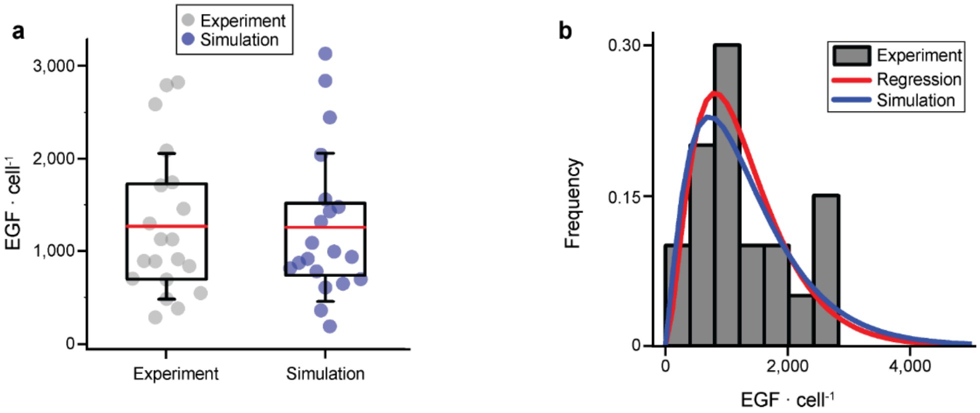

Supplementary Figure 10. Comparison between experiment and simulation for QD-EGF binding per cell. MDA-MB-231 cells were treated with QD-EGF (1 nM; EGF:QD = 0.3:1) at 37ºC for 5 min. Excess QD-EGF was washed away and cells were incubated at 37ºC for an additional 5 min before fixation. Simulations are described in **Methods**. **(a)** The number of QD-EGF bound per cell was not significantly different between experiment and stimulation ($p > 0.5$). The boxes indicate 25/75th percentile; red lines indicate mean value; whiskers are s.d. **(b)** Histogram shows the number of QD-EGF per cell measured experimentally. Gamma distributions were fit to experimental data (red line) and simulation data (blue line) using maximum likelihood estimation. Both the regression and simulation fit well to the experimental data, with p = 0.63 and 0.71, respectively, calculated using χ^2 tests**.**

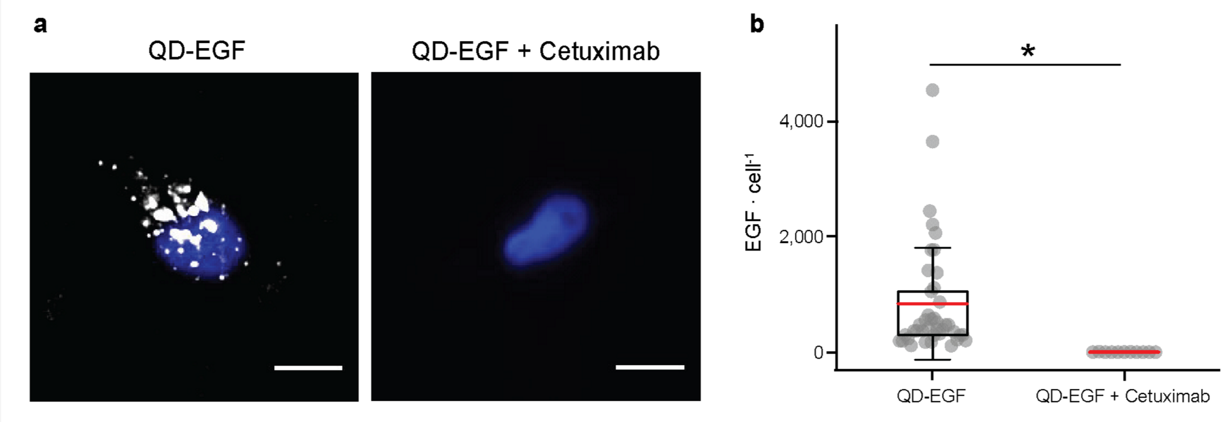

Supplementary Figure 11. **Binding specificity of QD-EGF to EGFR. (a)** Representative images of micro-patterned MDA-MB-231 treated with 1 nM QD-EGF (EGF:QD = 0.33) in the absence and presence of 20 nM Cetuximab at 37ºC for 5 min. Excess QD-EGF was washed away and cells were incubated at 37ºC for an additional 25 min in the absence and presence of 20 nM Cetuximab before fixation. Scale bar, 10 µm. **(b)** Distribution of EGF number per cell for conditions in (a). Boxes indicate 25/75th percentile; red lines indicate mean values; whiskers are s.d. Asterisks (*) indicates $p < 0.05$ and N.S. indicates not significant ($p > 0.05$).

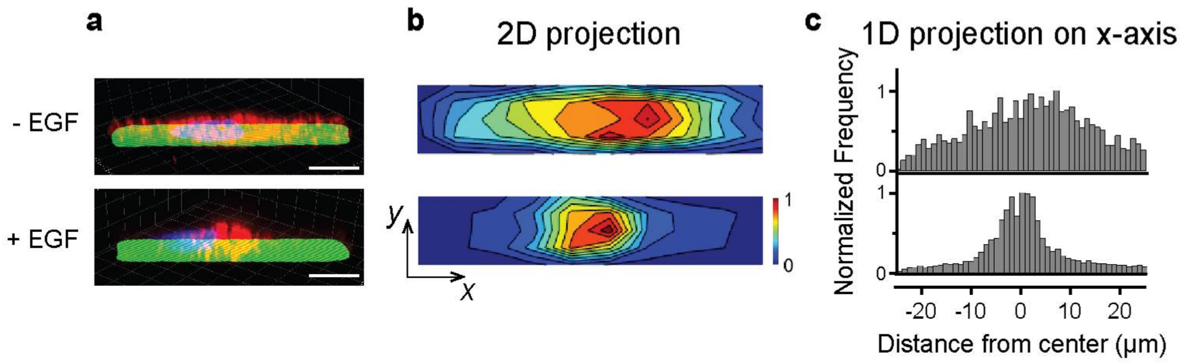

Supplementary Figure 12. **EGFR spatial distribution. (a)** Representative 3D images of MDA-MB-231 cells in the absence or presence of 100 ng/mL free EGF at 37ºC for 30 min. Immunofluorescent stain of EGFR is shown in red, nuclei are blue, and Alexa488-conjugated fibronectin micropatterns are green. **(b)** 2D *z*-projections on *xy* fibronectin micropattern planes and **(c)** 1D projections on *x*-axes indicate the averaged localization of EGFR on the cells in panel (a).

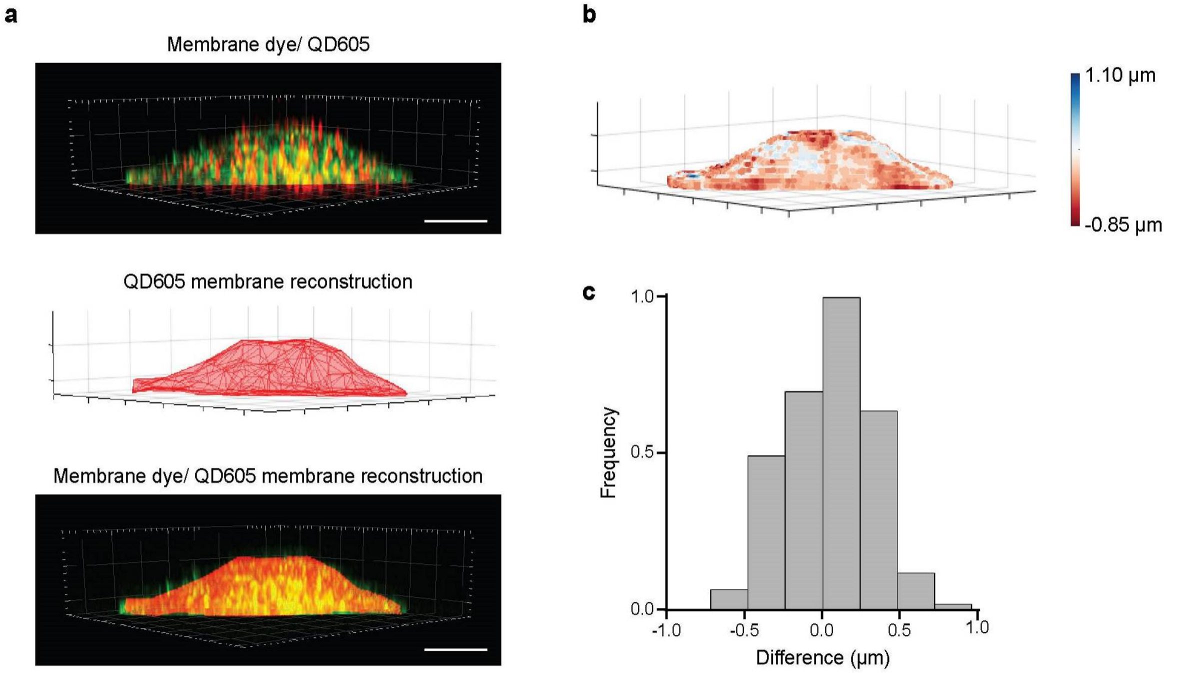

Supplementary Figure 13. **Comparison between 3D membrane map constructed from volumetric confocal images of membrane dyes and epifluorescence images of QD-labeled receptors. (a)** Overlay of raw 3D images of membrane dye (green) and QD605-IgG conjugates labels (red) on the same cell (top). The membrane dye images were acquired with confocal microscopy while QD605 images were acquired with epifluorescence microscopy as described in **Methods**. The 3D membrane map was reconstructed as alpha shapes from the QD605 images (middle). The bottom image shows the overlay of the 3D membrane dye image (green) with the reconstructed alpha shape map (red). Scale bar, 10 μm. **(b)** 3D map of differences between the two images. **(c)** Distribution of differences between membrane locations acquired from the two membrane maps.

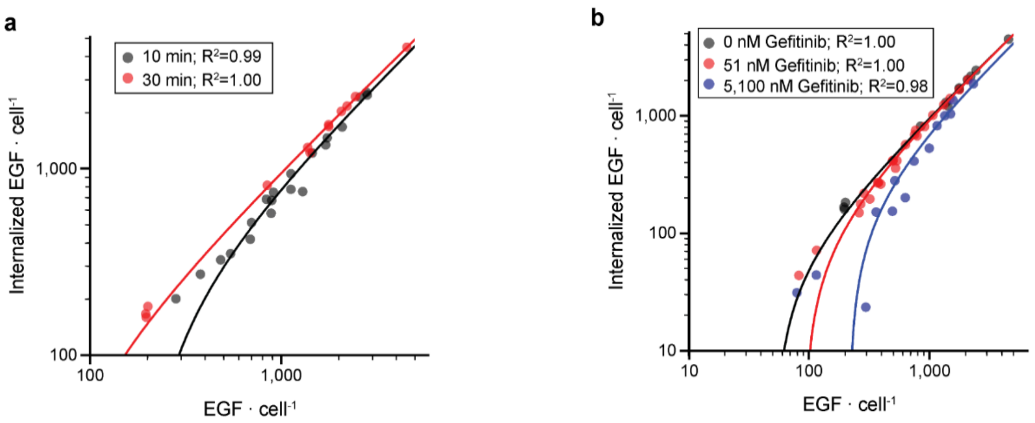

Supplementary Figure 14. **Correlation between EGF bound and EGF internalized in MDA-MB-231 cells.** Log-log plots show **(a)** 10 and 30 min after EGF stimulation and **(b)** 30 min after EGF stimulation in the presence of inhibitor gefitinib at 0, 51, and 5,100 nM concentration. Linear regressions were fit in linear scale with R^2 values as indicated.

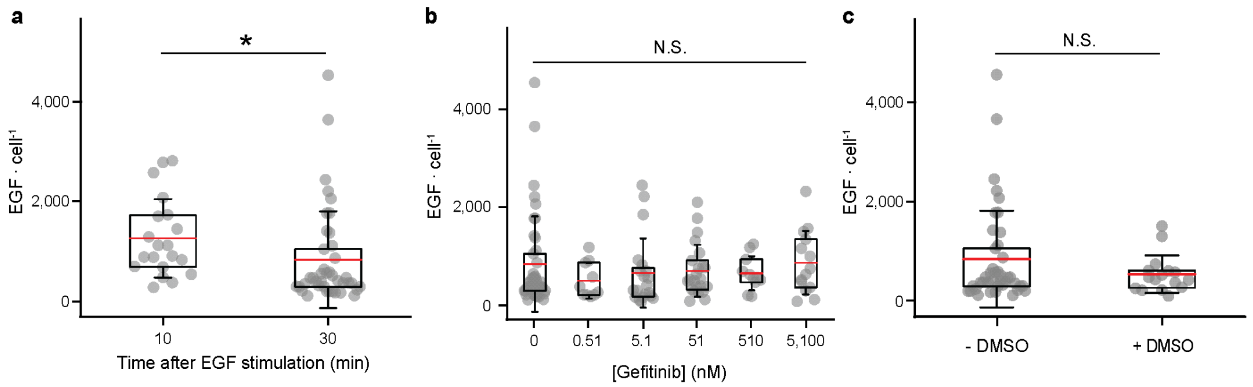

Supplementary Figure 15. **Distribution of EGF number per cell for conditions in Fig. 5.** Plots show **(a)** different times after stimulation, **(b)** different gefitinib concentrations, and **(c)** the impact of DMSO. Boxes indicate 25/75th percentile; red line indicate mean value; whiskers are s.d. Asterisks (*) indicates $p < 0.05$ and N.S. indicates not significant ($p > 0.05$).

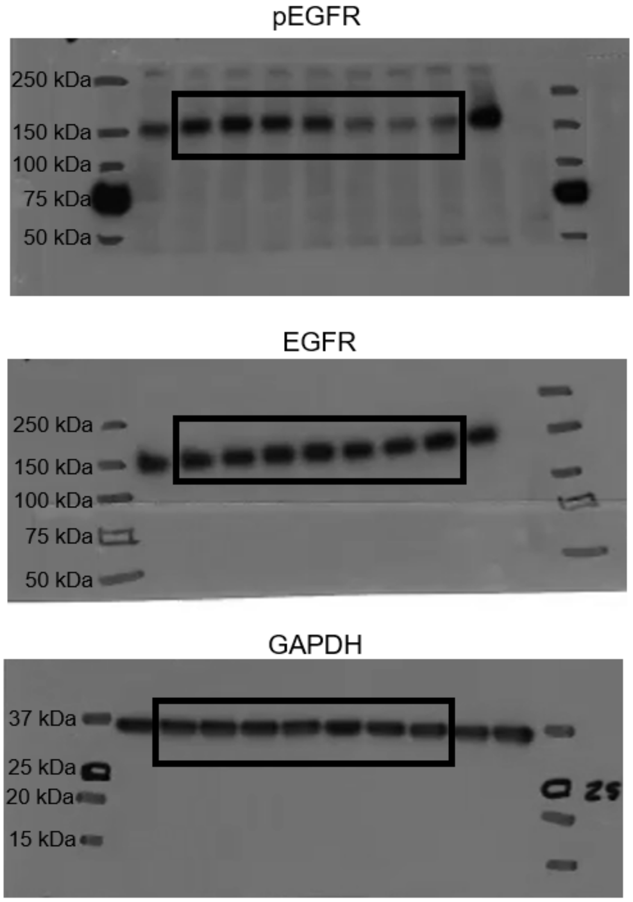

Supplementary Figure 16. **Uncropped western blots.** Black rectangles indicate cropped areas in Figure 5f.

Supplementary Table 1. Gamma distribution parameters for experiment and simulation

[QD-EGF]	T	a_{exp}	b_{exp}	a_{sim_1}	b_{sim_1}	a_{sim_2}	b_{sim_2}
1 nM	37ºC	2.8	460	3.3	394	3.3	396
0.1 nM	4ºC	1.1	7.6	3.3	2.7	2.4	4.0
1 nM	4ºC	2.6	31	3.3	27	3.2	28
10 nM	4ºC	2.2	440	3.3	265	3.3	266

T: Temperature
a_{exp}, b_{exp}: Fit to experimental data by Maximum Likelihood Estimate
a_{sim_1}, b_{sim_1}: Fit to extrinsic noise simulation by Maximum Likelihood Estimate
a_{sim_2}, b_{sim_2}: Fit to extrinsic and intrinsic noise simulation by Least Squares method

Supplementary References

1 Sheung, J. Y. et al. Structural Contributions to Hydrodynamic Diameter for Quantum Dots Optimized for Live-Cell Single-Molecule Tracking. *J. Phys. Chem. C* **122**, 17406-17412 (2018).
2 Klumperman, J. & Raposo, G. The complex ultrastructure of the endolysosomal system. *Cold Spring Harb. Perspect. Biol.* **6**, a016857 (2014).
