# Supplementary material for: Counting growth factors in single cells with infrared quantum dots to measure discrete stimulation distributions
Source: Nat Commun. 2019 Feb 22;10:909. doi: 10.1038/s41467-019-08754-5 (PMC6385258; doi:10.1038/s41467-019-08754-5)
Supplement: Supplementary file 2 — Reporting Summary [file 41467_2019_8754_MOESM2_ESM.pdf]

natureresearch

Corresponding author(s): Andrew M. Smith

Last updated by author(s): Jan 14, 2019

Reporting Summary

Nature Research wishes to improve the reproducibility of the work that we publish. This form provides structure for consistency and transparency in reporting. For further information on Nature Research policies, see Authors & Referees and the Editorial Policy Checklist.

Statistics

For all statistical analyses, confirm that the following items are present in the figure legend, table legend, main text, or Methods section.

n/a	Confirmed	
☐	☒	The exact sample size (n) for each experimental group/condition, given as a discrete number and unit of measurement
☐	☒	A statement on whether measurements were taken from distinct samples or whether the same sample was measured repeatedly
☐	☒	The statistical test(s) used AND whether they are one- or two-sided<br>*Only common tests should be described solely by name; describe more complex techniques in the Methods section.*
☒	☐	A description of all covariates tested
☒	☐	A description of any assumptions or corrections, such as tests of normality and adjustment for multiple comparisons
☐	☒	A full description of the statistical parameters including central tendency (e.g. means) or other basic estimates (e.g. regression coefficient) AND variation (e.g. standard deviation) or associated estimates of uncertainty (e.g. confidence intervals)
☐	☒	For null hypothesis testing, the test statistic (e.g. F, t, r) with confidence intervals, effect sizes, degrees of freedom and P value noted<br>*Give P values as exact values whenever suitable.*
☒	☐	For Bayesian analysis, information on the choice of priors and Markov chain Monte Carlo settings
☒	☐	For hierarchical and complex designs, identification of the appropriate level for tests and full reporting of outcomes
☒	☐	Estimates of effect sizes (e.g. Cohen's d, Pearson's r), indicating how they were calculated

Our web collection on statistics for biologists contains articles on many of the points above.

Software and code

Policy information about availability of computer code

Data collection	Zeiss Zen software
Data analysis	Matlab R2018a (MathWorks), Origin 2018 (Origin Lab), Autoquant X3 (Media Cybernetics), Imaris 9.2 (Bitplane)

For manuscripts utilizing custom algorithms or software that are central to the research but not yet described in published literature, software must be made available to editors/reviewers. We strongly encourage code deposition in a community repository (e.g. GitHub). See the Nature Research guidelines for submitting code & software for further information.

Data

Policy information about availability of data

All manuscripts must include a data availability statement. This statement should provide the following information, where applicable:

- Accession codes, unique identifiers, or web links for publicly available datasets
- A list of figures that have associated raw data
- A description of any restrictions on data availability

The data that support the findings of this study are available from the corresponding author upon reasonable request.

Field-specific reporting

Please select the one below that is the best fit for your research. If you are not sure, read the appropriate sections before making your selection.

☒ Life sciences ☐ Behavioural & social sciences ☐ Ecological, evolutionary & environmental sciences

For a reference copy of the document with all sections, see nature.com/documents/nr-reporting-summary-flat.pdf

Life sciences study design

All studies must disclose on these points even when the disclosure is negative.

Sample size	Samples sizes were selected to show a representative distribution of cells studied based on initial study of EGF binding distribution at multiple concentration of EGF. No sample-size calculation was performed, as the goal was to measure a distribution rather than a mean.
Data exclusions	For flow cytometry, dead cells and multiple-cell clusters were excluded to analyze single cells.<br>For imaging, dividing cells and dead cells are excluded.
Replication	All attempts at replication were successful, and a replicate data set is shown in the manuscript.
Randomization	No randomization was performed.
Blinding	Investigators were not blinded.

Reporting for specific materials, systems and methods

We require information from authors about some types of materials, experimental systems and methods used in many studies. Here, indicate whether each material, system or method listed is relevant to your study. If you are not sure if a list item applies to your research, read the appropriate section before selecting a response.

Materials & experimental systems

n/a	Involved in the study	
☐	☒	Antibodies
☐	☒	Eukaryotic cell lines
☒	☐	Palaeontology
☒	☐	Animals and other organisms
☒	☐	Human research participants
☒	☐	Clinical data

Methods

n/a	Involved in the study	
☒	☐	ChIP-seq
☐	☒	Flow cytometry
☒	☐	MRI-based neuroimaging

Antibodies

Antibodies used	Mouse anti-human EGFR antibody (BD Biosciences, cat. no. 555996) was used for immunofluorescence. Mouse anti-human EGFR antibody (Abcam, cat. no. ab32077), rabbit anti-human pEGFR antibody (R&D, MAB3570) and rabbit anti-human GAPDH (Cell Signaling) were used for western blot.
Validation	All antibodies were validated through either western blot-determined specificity to the antigen with known molecular weight or by distinguishable staining pattern by immunofluorescence.

Eukaryotic cell lines

Policy information about cell lines

Cell line source(s)	MCF-7 cells (ATCC, HTB-22), MDA-MB-231 cells (ATCC, HTB-26), or MDA-MB-468 cells (ATCC, HTB-132)
Authentication	Cell lines are authenticated by ATCC
Mycoplasma contamination	Cell lines were not tested for mycoplasma contamination
Commonly misidentified lines<br>(See ICLAC register)	None

Flow Cytometry

Plots

Confirm that:

☒ The axis labels state the marker and fluorochrome used (e.g. CD4-FITC).

☒ The axis scales are clearly visible. Include numbers along axes only for bottom left plot of group (a 'group' is an analysis of identical markers).

☒ All plots are contour plots with outliers or pseudocolor plots.

☒ A numerical value for number of cells or percentage (with statistics) is provided.

Methodology

Sample preparation	MDA-MB-231 cells (ATCC, HTB-26) were seeded in a T-75 cell culture flask in DMEM supplemented with 10% FBS and cultured until 90% confluence. Cells were washed once with PBS and treated with 5 mL Accutase at room temperature until fully detached from the surface. Accutase was removed by centrifugation for 5 min at 200xg and cells were washed once with ice-cold PBS containing 0.5% BSA and resuspended in the same medium at 3 x 10^6 cells mL^-1. Cell suspensions were then mixed in equal volume (25 µL) with ice-cold solutions of QD-EGF (0.06 – 120 nM; EGF:QD = 0.33) or dye-EGF (0.02 – 40nM). Control samples to measure non-specific binding were prepared identically but 2 µM unlabeled EGF. The cells were incubated at 4ºC for 4 hr with rocking, washed 3 times with ice-cold PBS containing 0.5% BSA, and resuspended in PBS.
Instrument	BD Biosciences LSR Fortessa Cytometry Analyzer equipped with 488 nm and 561 nm lasers in the Roy J. Carver Biotechnology Center at the University of Illinois.
Software	Data were collected using BDBiosciences software and analyzed using FCS Express 5
Cell population abundance	A minimum of 10,000 single cells was measured for each condition.
Gating strategy	Single cells were selected using a forward scatter width gate.

☒ Tick this box to confirm that a figure exemplifying the gating strategy is provided in the Supplementary Information.
